# Brain-inspired chaotic spiking backpropagation

**DOI:** 10.1093/nsr/nwae037

**Published:** 2024-01-30

**Authors:** Zijian Wang, Peng Tao, Luonan Chen

**Affiliations:** Key Laboratory of Systems Health Science of Zhejiang Province, School of Life Science, Hangzhou Institute for Advanced Study, University of Chinese Academy of Sciences, Hangzhou 310024, China; Key Laboratory of Systems Health Science of Zhejiang Province, School of Life Science, Hangzhou Institute for Advanced Study, University of Chinese Academy of Sciences, Hangzhou 310024, China; Key Laboratory of Systems Health Science of Zhejiang Province, School of Life Science, Hangzhou Institute for Advanced Study, University of Chinese Academy of Sciences, Hangzhou 310024, China; Key Laboratory of Systems Biology, Shanghai Institute of Biochemistry and Cell Biology, Center for Excellence in Molecular Cell Science, Chinese Academy of Sciences, Shanghai 200031, China; Guangdong Institute of Intelligence Science and Technology, Hengqin, Zhuhai 519031, China; Pazhou Laboratory (Huangpu), Guangzhou 510555, China

**Keywords:** spiking neural networks, surrogate gradient, local minima, backpropagation, brain-inspired learning, chaos

## Abstract

Spiking neural networks (SNNs) have superior energy efficiency due to their spiking signal transmission, which mimics biological nervous systems, but they are difficult to train effectively. Although surrogate gradient-based methods offer a workable solution, trained SNNs frequently fall into local minima because they are still primarily based on gradient dynamics. Inspired by the chaotic dynamics in animal brain learning, we propose a chaotic spiking backpropagation (CSBP) method that introduces a loss function to generate brain-like chaotic dynamics and further takes advantage of the ergodic and pseudo-random nature to make SNN learning effective and robust. From a computational viewpoint, we found that CSBP significantly outperforms current state-of-the-art methods on both neuromorphic data sets (e.g. DVS-CIFAR10 and DVS-Gesture) and large-scale static data sets (e.g. CIFAR100 and ImageNet) in terms of accuracy and robustness. From a theoretical viewpoint, we show that the learning process of CSBP is initially chaotic, then subject to various bifurcations and eventually converges to gradient dynamics, consistently with the observation of animal brain activity. Our work provides a superior core tool for direct SNN training and offers new insights into understanding the learning process of a biological brain.

## Teaser

The chaotic spiking backpropagation algorithm, drawing inspiration from the chaotic dynamics observed in the brain, has been utilized to directly train spiking neural networks, exhibiting superior performance compared with the current state-of-the-art methods.

## INTRODUCTION

Artificial neural networks (ANNs) or deep neural networks have been widely used in many fields, including image identification [1–4], machine translation [5,6], protein structure prediction [7] and gaming [8,9]. However, with the increasing size of ANNs, the burden of computing resources and energy grows [10]—that is, the great success of ANNs comes at the cost of massive large-scale computation and high energy consumption. Furthermore, ANNs remain divergent from real human brains from structural and energy-consuming viewpoints. To overcome these shortcomings by mimicking natural neural networks, spiking neural networks (SNNs) [11] have been proposed as the next generation of ANNs, and are attracting increasing interest from the research community. Compared with the continuous output of ANNs, SNNs utilize binary values for output: 1 for a spiking event and 0 for no signal. Moreover, in SNNs, information is transmitted as an event-driven spiking signal. The neurons are active only when receiving or emitting spikes. Due to such high spike sparsity and simple operations in the network, SNNs have more significant advantages than ANNs from both biologically plausible [12] and energy-consuming [13] aspects, so they are currently applied in neuromorphic hardware [14]. A recent study shows that SNNs consume only 0.02% of the energy of ANNs, whose efficiency is comparable to that of the human brain [15].

Nevertheless, unlike backpropagation (BP) [16] in training ANNs, the spiking function in SNNs is discontinuous and non-differentiable. Thus, it is a challenging task to train SNNs directly. Except for unsupervised learning algorithms, such as the Hebbian learning rule and Spike-Timing-Dependent Plasticity, the most popular SNN training methods were developed from ANNs and can be divided into two classes: the ANN-to-SNN conversion method [17] and the surrogate gradient (SG) method [18,19]. The conversion approach is to convert a pretrained ANN to an SNN with the same architecture and the hyperparameters of the SNN (leak-time constants, refractory period and membrane threshold). Specifically, ANN-to-SNN conversion first trains an ANN with rectified linear units and then replaces them with spiking neurons using a few conservation schemes (e.g. normalization, coding and reweighting). The converted SNNs can perform as well as the original ANNs on image classification of static data sets [20,21], including CIFAR10, CIFAR100 and ImageNet. In contrast, SNNs trained this way often have higher latency and energy consumption than directly trained SNNs [22]. Furthermore, the ANN-to-SNN method cannot capture the temporal information of SNNs, so it is often inaccurate in image classification of neuromorphic data sets, such as DVS-CIFAR10 and DVS-Gesture. In contrast, the SG method introduces backpropagation through time (BPTT) for training recurrent neural networks (RNNs) into SNNs using the gradient of a differentiable surrogate function and making it possible to directly train a large scale of SNN efficiently, such as CIFARNet [23], Spiking VGG [24] and Spiking ResNet [24,25]. However, the SG strategy makes the gradient prone to exploding or vanishing, leading to network degradation [26]. In addition, the SG method can only approximate the true gradient, and this approximation bias degrades the performance of the trained model. Therefore, developing an effective method for efficient learning of SNNs while keeping their original brain-like advantages is strongly needed.

In contrast to the gradient dynamics used in the BP algorithm of current deep learning, Skarda and Freeman's experiments in 1987 showed that the chaotic dynamics of brain neurons is indispensable to rabbits for acquiring new olfactory patterns [27]. Afterward, Matsumoto *et al.* found that the nervous system of a squid perceives external information using chaotic dynamics [28]. In addition, several recent experiments have also demonstrated that animal brain neural networks operate in a critical or quasi-critical state that lies between order and chaos [29]. These studies suggest that, with millions of years of evolution or screening, animal brains also exploit the chaotic dynamics of brain neurons for information processing. Inspired by this, Aihara *et al.* presented a chaotic neural network [30] by introducing biologically plausible chaos into conventional neural networks. By exploring the ergodicity and pseudo-randomness of chaotic dynamics, Chen and Aihara further proposed a transient chaotic neural network (TCNN) by designing chaotic simulated annealing (CSA), which significantly improves the global optimization performance in combinatorial optimization problems [31]. After that, many chaotic optimization algorithms were developed and a large body of work has shown that chaotic dynamics is very effective for global optimization problems [32–34], such as channel assignment for acellular mobile communications [35] and control of automatic regulator voltage [36]. Recently, Tao *et al.* integrated this chaos into a BP algorithm to create chaotic backpropagation (CBP) [37]. They successfully applied it to the learning or training process of multi-layer perceptions (MLPs), in which the chaotic dynamics significantly boosted the optimization and generalization performance of deep learning. Since SNNs are inspired by biological nervous systems unlike traditional ANNs, it is natural to assume that such chaotic dynamics of the brain could make SNN learning much more efficient.

This paper introduces the brain-like chaotic dynamics by adding negative feedback to each neuron in the training process of SNNs and proposes the chaotic spiking backpropagation (CSBP) method as a general core algorithm for SNN training, whose implementation is simple and robust. From a computational viewpoint, we show that CSBP outperformed the existing state-of-the-art (SOTA) methods on traditional static large-scale data sets (e.g. ImageNet and CIFAR100) and neuromorphic data sets (e.g. DVS-CIFAR10 and DVS-Gesture). Specifically, by exploiting the ergodicity and pseudo-randomness of chaotic dynamics, CSBP reaches a lower training loss and performs better generalization (testing) on benchmark data sets. From a theoretical viewpoint, we show mathematically that the learning process of CSBP is chaotic during the initial stage. Subject to various bifurcations and eventually converging to gradient dynamics, this is consistent with the observation of animal brain activity. The results demonstrate that CSBP not only provides a powerful core tool for SNN training as a general and robust algorithm, but also offers new insights into understanding the learning mechanisms of the real brain.

## RESULTS

In this section, we first introduce the overall framework of CSBP, examine the learning behavior of CSBP on small SNN models, then compare the CSBP performances on large-scale benchmark data sets for SNN learning, and finally discuss the robustness and computational complexity of CSBP.

### CSBP algorithm for SNN

**Figure 1.**
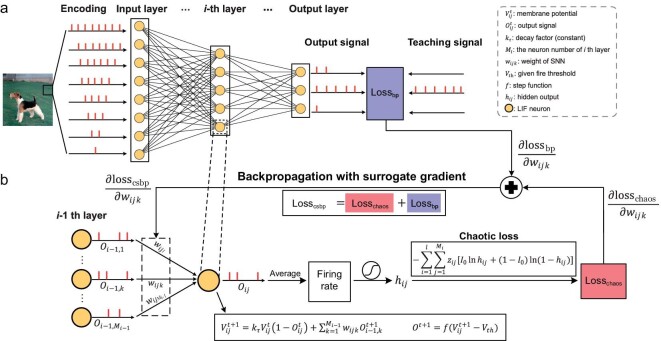
BP and CSBP algorithms in SNN models. (a) A schematic diagram of a (common) gradient-based BP algorithm that learns the weights based on the difference between the output signal and the teaching signal, or the loss function ${{\rm los}}{{{\rm s}}}_{{\mathrm{bp}}}$. (b) In contrast to the common BP algorithm in (a), the chaotic spiking backpropagation (CSBP) algorithm introduces an additional brain-inspired loss function, ${{\rm los}}{{{\rm s}}}_{{\mathrm{chaos}}}$, which comes from the output of each neuron. Specifically, CSBP learns the weights by backpropagating the surrogate gradient of ${{\rm los}}{{{\rm s}}}_{{\mathrm{csbp}}} = {{\rm los}}{{{\rm s}}}_{{\mathrm{bp}}} + {{\rm los}}{{{\rm s}}}_{{\mathrm{chaos}}}$ to the weights. Such a slight difference due to ${{\rm los}}{{{\rm s}}}_{{\mathrm{chaos}}}$ enables the algorithm to generate chaotic dynamics both theoretically and numerically. From a dynamical perspective of each neuron, it is equivalent to introducing negative feedback for updating dynamics of each weight. The meanings of the symbols are given in the dashed line box.

An SNN is composed of neurons that transmit and process the information on the occurrence of a spike or an event generated by a neuron, which is the central computing unit. Among the many neural models, the leaky integrate-and-fire (LIF) model is widely used because it considers both the simulation accuracy and speed of neural dynamics. Here, to describe the CSBP method, we utilize the LIF model (Fig. 1a). It should be noted that other neuronal models of SNNs can also be applied similarly. Specifically, we adopt a simplified first-order differential equation of the LIF model with the Euler method [23]; its update rule can be written as follows:


(1) \begin{eqnarray*}
{V_{ij}^{t + 1} = {k}_\tau V_{ij}^t\Big( {1 - O_{ij}^t} \Big) + \mathop \sum \limits_{k = 1}^{{M}_{i - 1}} {w}_{ijk}O_{i - 1,k}^{t + 1}}\\ {O_{ij}^{t + 1} = f\Big( {V_{ij}^{t + 1} - {V}_{th}} \Big)},
\end{eqnarray*}

where $V_{ij}^t$ and $O_{ij}^t\ $are the membrane potential and the output signal of the *j*-th spiking neuron in the *i*-th layer at time *t* ($j = 1,2, \ldots ,{M}_i;i = 1,2, \ldots ,l;t = 1,2, \ldots ,T$), respectively; ${k}_\tau $ is a constant called the decay factor; ${w}_{ijk}$ is the weight from the *k*-th neuron in the ($i - 1$)-th layer to the *j*-th neuron in the *i*-th layer; and ${V}_{th}$ is the given fire threshold (Fig. 1a). $f( x )$ is the step function, which reaches $0$ when $x < 0$ and 1 otherwise. ${M}_i$ is the number of neurons in the *i*-th layer and *T* is the simulation time.

To introduce biologically plausible chaotic dynamics in an SNN, a chaotic loss, ${\mathrm{los}}{{\mathrm{s}}}_{\\smallriptstyle{\rm chaos}}$, was added to the original training loss, ${\mathrm{los}}{{\mathrm{s}}}_{\rm bp}$, which could be in any form, such as the mean square error (MSE) or cross-entropy (Fig. 1b):


(2) \begin{eqnarray*}
{\mathrm{los}}{{\mathrm{s}}}_{{\mathrm{chaos\ }}}\! =\! {-} \mathop \sum \limits_{i = 1}^l \mathop \sum \limits_{j = 1}^{{M}_i} {z}_{ij}[ {{I}_0\ln {h}_{ij}\! +\! ( {1\! -\! {I}_0} )\ln ( {1\! -\! {h}_{ij}} )} ],\!\!\!\!\!\\\end{eqnarray*}

where


(3) \begin{eqnarray*}
{h}_{ij} &=& {\mathrm{sigmoi}}{{\mathrm{d}}}\!\left( {\frac{1}{T}\mathop \sum \limits_{t = 1}^T \mathop \sum \limits_{k = 1}^{{M}_{i - 1}} {w}_{ijk}O_{i - 1, k}^t} \right)\\
&=& \frac{1}{{1 + \exp\! \left( { - \frac{1}{T}\mathop \sum \nolimits_{t = 1}^T \mathop \sum \nolimits_{k = 1}^{{M}_{i - 1}} {w}_{ijk}O_{i - 1,\ k}^t} \right)}}.\\
\end{eqnarray*}

Here, ${z}_{ij}$ is a scalar hyperparameter (or temperature parameter in an annealing process) for CSBP indicating the chaos intensity/strength for different ${w}_{ijk}$, and ${I}_0$ is a constant between 0 and 1 (we set it to 0.65 in all computations). Theoretically, this model can always generate chaos (e.g. Marotto chaos [38]) when ${I}_0 \ne 0.5$; see Note S5 and Fig. S3. Obviously, the gradient of ${\mathrm{los}}{{\mathrm{s}}}_{\\smallriptstyle{\rm chaos}}$ with respect to ${w}_{ijk}$ is:


(4) \begin{eqnarray*}
\frac{{\partial {\mathrm{los}}{{\mathrm{s}}}_{{\mathrm{chaos}}}}}{{\partial {w}_{ijk}}} &=& \frac{{\partial {\mathrm{los}}{{\mathrm{s}}}_{{\mathrm{chaos}}}}}{{\partial {h}_{ij}}}\frac{{\partial {h}_{ij}}}{{\partial {w}_{ijk}}}\\
&=& {-} {z}_{ij}\frac{{{I}_0 - {h}_{ij}}}{{{h}_{ij}(1 - {h}_{ij})}}{h}_{ij}(1 - {h}_{ij})\frac{1}{T}\sum\limits_{t = 1}^T {O_{i - 1,k}^t} \\
&=& {-} {z}_{ij}({I}_0 - {h}_{ij})\frac{1}{T}\sum\limits_{t = 1}^T {O_{i - 1,k}^t}.
\end{eqnarray*}

By summing over ${\mathrm{los}}{{\mathrm{s}}}_{\rm bp}$ and ${\mathrm{los}}{{\mathrm{s}}}_{\\smallriptstyle{\rm chaos}}$, we obtain the integrated loss function of CSBP—that is, ${\mathrm{los}}{{\mathrm{s}}}_{\\smallriptstyle{\rm csbp}} = {\mathrm{los}}{{\mathrm{s}}}_{\rm bp} + {\mathrm{los}}{{\mathrm{s}}}_{\\smallriptstyle{\rm chaos}}$, where ${\mathrm{los}}{{\mathrm{s}}}_{\rm bp}$ is the loss function of the traditional BP method. Therefore, in the scheme of standard gradient descent, the updating formula of ${w}_{ijk}$ for all $( {i,\ j,\ k} )$ in CSBP can be expressed as follows:


(5) \begin{eqnarray*}
&&\!\!{w}_{ijk} \leftarrow {w}_{ijk} - \eta \frac{{\partial {\mathrm{los}}{{\mathrm{s}}}_{\\smallriptstyle{\rm csbp}}}}{{\partial {w}_{ijk}}}\\
&&\!\! = {w}_{ijk} - \eta \frac{{\partial {\mathrm{los}}{{\mathrm{s}}}_{\rm bp}}}{{\partial {w}_{ijk}}} - \eta \frac{{\partial {\mathrm{los}}{{\mathrm{s}}}_{\\smallriptstyle{\rm chaos}}}}{{\partial {h}_{ij}}}\frac{{\partial {h}_{ij}}}{{\partial {w}_{ijk}}}\\
&&\!\! = {w}_{ijk} - \eta \frac{{\partial {\mathrm{los}}{{\mathrm{s}}}_{\rm bp}}}{{\partial {w}_{ijk}}} + \eta {z}_{ij}( {{I}_0 - {h}_{ij}} )\frac{1}{T}\mathop \sum \limits_{t = 1}^T O_{i - 1, k}^t, \\
\end{eqnarray*}

where $\eta $ is the learning rate. From a dynamical viewpoint, the last term (or chaotic term) at the right side of Equation (5) is equivalent to negative feedback (specific to ${h}_{ij}$, while ${I}_0$ is treated as a bias term) to the weights, which is introduced internally (or intrinsically) from the neuron itself, not externally from an unrelated system. Clearly, Equation (5) is simply a gradient BP without the last term. For each ${w}_{ijk}$ at the $( {m + 1} )$-th updating iteration, we can represent the updating of Equation (5) as a difference equation with updating iteration *m* as the pseudo-time, i.e.:


(6) \begin{eqnarray*}w_{ijk}^{m + 1} = {g}_{ijk}\!\left( {{W}^m} \right)\quad {\mathrm{ or }}\quad {W}^{m + 1} = G\!\left( {{W}^m} \right),\end{eqnarray*}

where ${g}_{ijk}( {{W}^m} )$ is the right side of Equation (5), ${W}^m$ is a vector of $w_{ijk}^m$ for all $( {i,\ j,\ k} )$ and *G* is a vector function of ${g}_{ijk}$ for all $( {i,\ j,\ k} )$; note that ${\mathrm{los}}{{\mathrm{s}}}_{\rm bp}$, ${h}_{ij}$ and $O_{i - 1,k}^t$ are all functions of ${W}^m$ with $m = 0,\ 1,\ 2,\ \ldots $. Thus, we can use dynamical systems theory to study the dynamics of Equation (5) for all $( {i,\ j,\ k} )$ with *m* as the time. Mathematically, we can prove that Equation (5) is not gradient dynamics but generates Marotto chaos when ${z}_{ij}$ is sufficiently large according to Theorem 1 and Theorem 2 (see Note S2 for the details and proof) [39–41] and its chaotic behaviors will also be numerically validated using a positive maximum Lyapunov exponent in the next section. Note that the main difference between ${\mathrm{los}}{{\mathrm{s}}}_{\\smallriptstyle{\rm chaos}}$ in Equation (2) and that in CBP is the output of neurons of the previous layer in the sigmoid function.

Theorem 1: Assume $1 > {I}_0 > 0$, and *z* is sufficiently large. Then *W* of Equation (6) has a unique fixed point that is a repeller $\bar{W}\ $with each element as:

\begin{eqnarray*}
{\bar{w}}_{ijk}\! =\! {\varepsilon }_{ik}{\mathrm{ln}}\frac{{{I}_0}}{{1\! -\! {I}_0}}\! +\! \mathcal{O}\!\left( {\frac{1}{z}} \right) \quad {\mathrm{ for\ }}i,j,k = 1,2, \ldots ,
\end{eqnarray*}

where $\mathcal{O}( {\frac{1}{z}} )$ is a term with the order of $\frac{1}{z}$ when *z* is sufficiently large.

Theorem 2: Assume $1 > {I}_0 > 0$ with ${I}_0 \ne 0.5$, and *z* is sufficiently large. Then $\bar{W}$ of Equation (6) is a snap-back repeller, or has a transversal homoclinic orbit that generates chaotic dynamics in the sense of Marotto. In particular, the following point $\ {\bar{W}}^0$ with each element is:

\begin{eqnarray*}
&&\bar{w}_{ijk}^0 = {\varepsilon }_{ik}{\mathrm{ln}}\frac{{2{I}_0 - 1}}{{2 - 2{I}_0}} + \mathcal{O}\!\left( {\frac{1}{z}} \right) \mathrm{ for\ }{I}_0 > 0.5, \\
&& \mathrm{or }\bar{w}_{ijk}^0 = {\varepsilon }_{ik}{\mathrm{ln}}\frac{{2{I}_0}}{{1 - 2{I}_0}} + \mathcal{O}\left( {\frac{1}{z}} \right)\\
&&{\mathrm{\ for\ }}{I}_0 < 0.5,
\end{eqnarray*}

for *i, j, k* = 1,2,…, is on this homoclinic orbit. Here, $\mathcal{O}( {\frac{1}{z}} )$ is a term with the order of $\frac{1}{z}$ when *z* is sufficiently large.

Marotto chaos [38] is a topological chaos in a high-dimensional system, which can be viewed as a generalized Li–Yorke chaos in a 1D system. Marotto chaos in terms of structure is so complicated that the system has any *p*-periodic points and, at the same time, a scrambled set or an uncountable set containing no periodic points with topological transitivity.

Although the chaotic dynamics in Equation (5) has been proven to have global search capability due to the chaotic behavior [39–41], unlike local gradient search in the traditional BP, we need to anneal the chaotic strength ${z}_{ij}$ in order for the learning or training process to converge to a stable solution. Here, we use the same CSA scheme as TCNN or CBP, namely:


(7) \begin{eqnarray*}{z}_{ij} \leftarrow \beta {z}_{ij},\end{eqnarray*}

where $\beta $ is an annealing constant and $0 < \beta < 1$. For convenience, we set all ${z}_{ij}$ equal to a scalar variable *z* in this work; that is, we denote the initial value of ${z}_{ij}$ as $z_{ij}^0$ and set all $z_{ij}^0$ to the same value, ${z}^0$, in practice. Obviously, as the training proceeds, i.e. $z_{ij}^{m + 1} = \beta z_{ij}^m$, the chaotic intensity *z* gradually becomes smaller. When *z* tends to 0, the chaotic term (i.e. the last term) in Equation (5) can be neglected, and then CSBP clearly degenerates to the traditional BP method, thus ensuring the convergence of the training process (see Fig. S11 and Note S11 for an intuitive example). Similarly, we can add the last term of Equation (5) as a plug-in unit to any BP-based method to improve its performance and robustness. Two main points explain the working principle of CSBP. One is that CSBP always produces chaotic dynamics when intensity *z* is sufficiently large (Note S2) and, since chaos is ergodic (or with chaotic itinerancy) in its fractal space, it can usually sample a wider weight space. The other is that chaotic dynamics gradually transforms into gradient dynamics as *z* anneals, ensuring the convergence. Theoretically, CSBP has a global search ability as long as the chaotic dynamics samples the attractor region of the global optimal solution, and its numerical convergence to the global optimal solution is also validated when the annealing rate is sufficiently slow [40,41]. From the viewpoint of optimization, the chaotic dynamics generated by CSBP provides a better initial solution for the BP algorithm.

### Validation of CSBP on small SNN models

**Figure 2.**
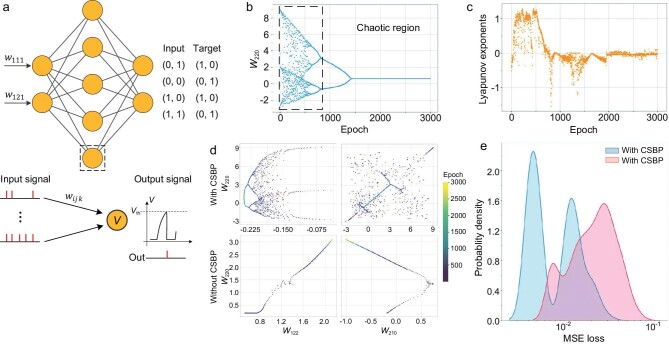
Global searching ability of CSBP on a small SNN. (a) The settings of the XOR task and a [2,5,2] SNN are shown above while the spiking pattern for each LIF neuron in the small SNNs is shown below. (b) With the chaotic strength decreasing, the weight ${w}_{220}$ in the SNN exhibits chaotic dynamics and then converges. The dashed box shows a chaotic region when chaotic strength is sufficiently large. (c) The maximum Lyapunov exponent of ${w}_{220}$ during the training in the XOR task. (d) The learning dynamic curves in the weight space (${w}_{220}$ and ${w}_{122}$, ${w}_{220}$ and ${w}_{210}$) while training with CSBP and without CSBP. (e) The probability density of MSE loss in the last 500 epochs out of 10 000 epochs in total.

Before applying CSBP to real data sets, we conducted experiments on small SNNs for the exclusive or (XOR) classification task and a simple regression task. We used an SNN model with the architecture of [2,5,2] and the training data in Fig. 2a. *z* and *β* were taken as 20 and 0.9995, respectively. The curve of one representative weight, ${w}_{220}$ (0 in the subscript index represents the bias), in the SNN and the corresponding Lyapunov exponents (Note S9) are shown in Fig. 2b and c, respectively. At the beginning of training (i.e. when *z* is sufficiently large, the dashed box in Fig. 2b), ${w}_{220}$ produces stochastic-like dynamics, which correspond to a positive Lyapunov exponent and imply that CSBP produces chaotic dynamics. In addition, we further compare the learning trajectories of BP and CSBP in a 2D weight space. As shown in the two examples in Fig. 2d, compared with the BP algorithm, the chaotic dynamics introduced by CSBP greatly improves the sampling efficiency of the model in the weight space. Finally, as the chaotic intensity *z* gradually decreases to 0, CSBP degenerates into the BP algorithm, at which point the MSE loss fluctuates in a small interval and converges. Figure 2e compares the distribution of the MSE loss after the convergence of CSBP and BP, and it can be found that, after the introduction of the chaotic dynamics of CSBP, the original loss is significantly reduced. In other words, chaos can effectively help the model jump out of local minima. Similar results can be obtained from a simple regression task (Note S6 and Fig. S4).

### CSBP improved the performance of SNNs on benchmarks

To illustrate the effectiveness of the CSBP method, we selected the directly trained SOTA methods of several SNNs as baseline methods and introduced the chaotic loss of CSBP on top of these methods (i.e. added the last term of Equation (5) as a plug-in unit) and then compared the performance of each model with and without CSBP on four mainstream data sets (DVS-CIFAR10 and DVS-Gesture as neuromorphic data sets; CIFAR100 and ImageNet as large-scale static data sets). It should be noted that, except for the two additional hyperparameters, ${z}^0$and${\mathrm{\ }}\beta $, all other hyperparameters (e.g. network architecture, optimizer and random seed) of CSBP were set the same as by their corresponding baseline (SOTA) method. In the following, we present the specific results for each of the four data sets.

For the DVS-CIFAR10 (event-stream) data set, which was specifically designed for SNN testing, we first adopted Wide-7B-Net with the baseline method of spike-element-wise (SEW) [25]. We introduced CSBP in the two down-sampling layers of Wide-7B-Net, and the corresponding learning curves with CSBP and without CSBP are shown in Fig. S5. The maximum test accuracies of CSBP at *T* = 4 and 8 are 73.5% and 74.9%, respectively, which are significantly higher than those of the original SEW method (64.8% and 70.2%, respectively) (Table 1). We also adopted a seven-layer CNN with the baseline method of temporal effective batch normalization (TEBN) [42]. After introducing chaotic dynamics to the first layer and linear layers, CSBP achieved a test accuracy of 81.7%, which was significantly higher than that of TEBN (75.1%).

**Table 1.** Performances on neuromorphic data sets: DVS-CIFAR10 and DVS-Gesture. We compare our method with recent SOTA or comparative direct training methods (ACC: accuracy).

Data set	Method	Network	*T*	ACC (%)
DVS-CIFAR10	STBP-tdBN [44]	Spiking ResNet-19	10	67.8
	Conv3D [45]	LIAF-Net	10	71.7
	LIAF [45]	LIAF-Net	10	70.4
	HP [46]	Spiking CNN	–	67.8
	SEW [25]	Wide-7B-Net	4	64.8
			8	70.2
	TEBN [42]	7-layer CNN	10	75.1
	**SEW + CSBP**	**Wide-7B-Net**	**4**	**73.5**
			**8**	**74.9**
	**TEBN + CSBP**	**7-layer CNN**	**10**	**81.7**
DVS-Gesture	BPTT [47]	CNN-based 8 layers	60	93.4
	STBP-tdBN [44]	Spiking ResNet-19	40	96.9
	HP [46]	Spiking CNN	–	97.0
	SEW [25]	7B-Net	16	97.9
	**SEW + CSBP**	**7B-Net**	**16**	**98.3**

For the DVS-Gesture data set, another popular neuromorphic data set, we adopted 7B-Net with the baseline method of SEW [25]. We introduced CSBP in the first down-sampling layer of 7B-Net. The corresponding learning curves with CSBP and without CSBP are shown in Fig. S6. The simulation time was set to 16. The difference in maximum test accuracies is shown in Table 1 (98.3% for CSBP and 97.9% for the baseline method).

**Figure 3.**
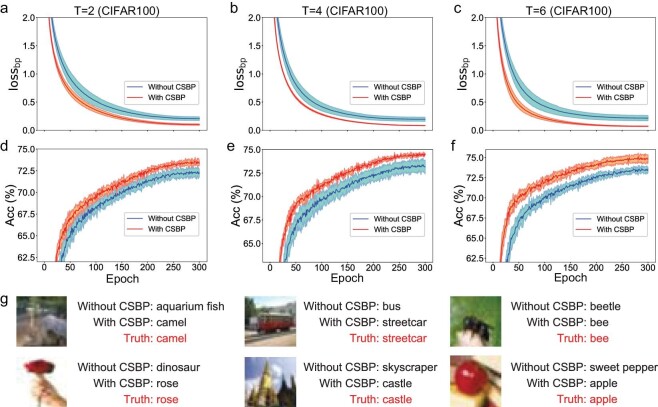
Comparison of CSBP and each baseline method on the CIFAR100 data set. The learning curves of BP loss, ${\mathrm{los}}{{\mathrm{s}}}_{\rm bp}$, on the training set, and the accuracies on the test set, are compared in (a)–(c) and (d)–(f), respectively. The three columns from left to right correspond to the results of CSBP and the baseline method for *T* = 2, 4 and 6, respectively. (g) shows six examples of the task of image classification, for which CSBP predicts correctly but the baseline method (i.e. without CSBP) does not.

For the CIFAR100 data sets, we adopted ResNet-19 [23] with the baseline method of temporal efficient training (TET) [43]. We used CSBP in the last spiking MLP layer in ResNet-19, and compared the performance on the training and test sets of CSBP and the baseline method (without CSBP). Figure 3a–f shows the change curves of BP loss (${\mathrm{los}}{{\mathrm{s}}}_{\rm bp}$) and test accuracy with CSBP and without CSBP under a simulation time period (*T*) of 2, 4 and 6. Clearly, the ${\mathrm{los}}{{\mathrm{s}}}_{\rm bp}$ of CSBP on the training set is lower than that of the corresponding baseline method regardless of *T*, while its accuracy on the test set is higher. To quantify the improvement of CSBP on the generalization performance of the baseline method, we calculated the maximum accuracy that the model could achieve on the test set during training at different *T*. As shown in Table 2, the maximum accuracies of the model for TET are 72.87%, 74.47% and 74.72% at *T* = 2, 4 and 6, respectively, while those of CSBP are 75.22%, 75.55% and 75.92%, respectively, which are higher than those of TET. Some examples of the image classification task that CSBP predicts correctly but current SOTA BP does not are shown in Fig. 3g. We also reproduced TEBN with data augmentation at *T* = 6 and achieved a test accuracy of 78.42%. When applying CSBP, we achieved a higher accuracy of 78.81% (Table 2).

**Table 2.** Performances on static data sets: ImageNet and CIFAR100. We compare our method with recent comparative direct training methods.

Data set	Method	Network	*T*	ACC (%)
ImageNet	STBP-tdBN [44]	Spiking ResNet-34	6	63.72
	PLIF [48]	Spiking ResNet-34	7	67.04
	SEW [25]	SEW ResNet-18	4	63.18
		SEW ResNet-34	4	67.04
	**SEW + CSBP**	**SEW ResNet-18**	**4**	**63.40**
		**SEW ResNet-34**	**4**	**67.62**
CIFAR100	IDE [49]	CIFARNet-F	100	73.07
	STBP-tdBN [44]	Spiking ResNet-19	2	69.41
			4	70.86
			6	71.12
	Diet-SNN [50]	Spiking ResNet-20	5	64.07
	TET [43]	Spiking ResNet-19	2	72.87
			4	74.47
			6	74.72
	TEBN [42]	Spiking ResNet-19	6	78.42
	**TET + CSBP**	**Spiking ResNet-19**	**2**	**75.22**
			**4**	**75.55**
			**6**	**75.92**
	**TEBN + CSBP**	**Spiking ResNet-19**	**6**	**78.81**

For the ImageNet data set, a large-scale data set, we selected SEW [25] as the baseline method combined with two SNN models: SEW ResNet-18 and SEW ResNet-34. We adopted CSBP in different residual blocks (see ‘Methods’) of SEW ResNet-18 and SEW ResNet-34. The corresponding learning curves of SEW and CSBP are shown in Fig. S7. The maximum test accuracies of the two models for CSBP were 63.40% and 67.62%, respectively, which are higher than those of the original SEW (63.18% and 67.04%, respectively, Table 2). We also selected TEBN as the baseline method combined with SEW ResNet-34 and achieved a test accuracy of 63.61%. When applying CSBP, we achieved a higher accuracy of 65.25% (see Fig. S8 for the learning curves with and without CSBP).

Overall, these results suggest that the chaotic dynamics in CSBP consistently improves the learning performance of SNNs and significantly contributes to the generalization performance of the baseline methods on various data sets including neuromorphic data sets (e.g. ≤9% for the DVS-CIFAR10 data set with $T = 4$).

### CSBP is computationally efficient and robust

**Figure 4.**
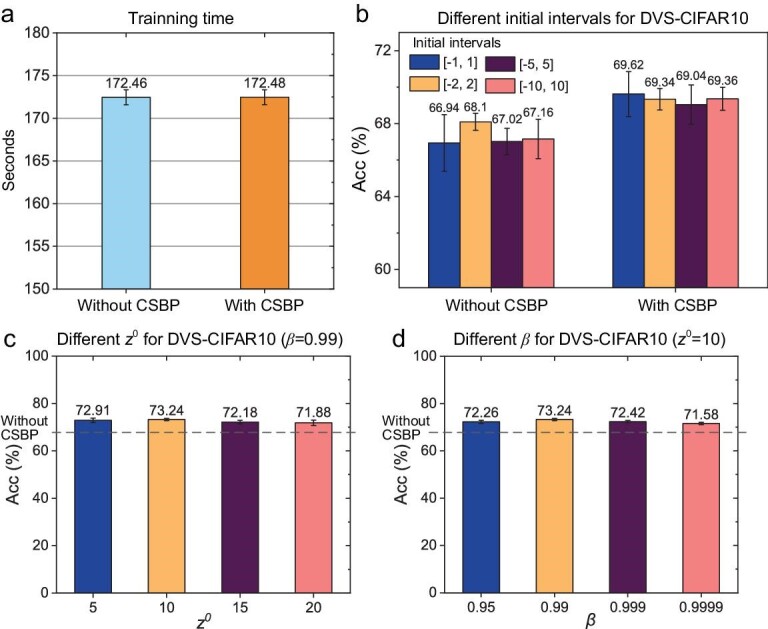
The comparison of training times on CIFAR100 and the robustness results with different hyperparameters and initial values for CSBP on DVS-CIFAR10. (a) The training time of each epoch of CSBP and the baseline method with *T* = 2 on Spiking-ResNet19. (b) Robustness on different initial conditions for CSBP and the baseline method. (c) Given $\beta = 0.99$, the average test accuracies of ${{\mathrm{z}}}^0 = 5,\ 10,\ 15\ {\mathrm{and}}\ 20$, respectively. (d) Given ${{\mathrm{z}}}^0 = 10$, the average test accuracies of $\beta = 0.95,\ 0.99,\ 0.999\ {\mathrm{and}}\ 0.9999$, respectively. Each result stands for the test accuracy in five independent experiments with different random seeds only.

In terms of computational cost, since only one loss term, ${\mathrm{los}}{{\mathrm{s}}}_{\\smallriptstyle{\rm chaos}}$, has been added, CSBP brings little extra time cost than the baseline method. As shown in Fig. 4a, we separately trained 150 epochs on CIFAR100 with the same hardware (NVIDIA V100 GPU) and test environment for both TET and TET with CSBP. The average training time per epoch spent by CSBP and TET was 172.48 and 172.46 seconds, respectively, which indicates that the computational costs of CSBP and TET are similar when the same iteration number is used.

An important feature of chaos is ergodicity, i.e. it samples the global optimum in the region of attractors whenever it undergoes sufficient dynamical evolution. This theoretically guarantees the robustness of the CSBP algorithm to the initial conditions and makes it significantly different from gradient-based methods. To validate CSBP computationally, we introduced chaotic dynamics to the two down-sampling layers in Wide-7B-Net [25] and trained the network on the DVS-CIFAR10 data set with different initial weights of the layers with CSBP (similarly in Table 2). Four different weight initial intervals were selected ($[ { - 1,\ 1} ]$, $[ { - 2,\ 2} ]$, $[ { - 5,\ 5} ]$ and $[ { - 10,\ 10} ]$). The distributions of the initial weights in the two down-sampling layers were uniform distributions in the intervals, and ${z}^0$ and $\beta $ were set to 10 and 0.9995, respectively. The chaotic intensity was annealed in every training batch. The results with five different random seeds in each case are shown in Fig. 4b. Generally, with different initial intervals, CSBP can improve the test accuracy on average. Moreover, with the four different initial intervals, the average test accuracies of CSBP are between 69% and 70%, which is more robust than the results without CSBP (between 66% and 69%). The results show that CSBP is robust on the initial conditions due to its chaotic dynamical features, alleviating the notorious local-minima problem of existing gradient-based BP algorithms.

In addition to the initial conditions, CSBP is also robust to the two hyperparameters, ${z}^0$ and $\beta $. As shown in Fig. 4c and d, we compared the results of different ${z}^0$ with the same $\beta $ and different $\beta $ with the same ${z}^0$ with *T* = 4 on DVS-CIFAR10. When setting $\beta = 0.99$, for CSBP with ${{\mathrm{z}}}^0 = 5,{\mathrm{\ }}10,{\mathrm{\ }}15{\mathrm{\ and\ }}20$, we obtained a test accuracy of 72.90%, 73.24%, 72.18% and 71.88%, respectively, on average (Fig. 4c, five independent experiments only with different random seeds, the same as below). When setting ${z}^0 = 10$, for CSBP with $\beta = 0.95, 0.99, 0.999{\mathrm{\ and\ }}0.9999$, we obtained a test accuracy of 73.24%, 72.42%, 71.58% and 70.36%, respectively, on average (Fig. 4d). Notably, the test accuracy was 64.8% for SEW without CSBP (Table 1). The above results show that the performance of SEW is consistently improved after introducing CSBP with different hyperparameters, indicating that CSBP is robust and hyperparameter-insensitive.

## DISCUSSION

SNNs could be a breakthrough for the bottlenecks of ANNs due to the advantages of energy consumption and the temporal code used in biological brains. However, it is still difficult to train SNNs in general, mainly owing to the local-minima problem of gradient-based BP, complex dynamics of neurons and the non-differentiable nature of spike operations [25]. In this work, replacing gradient search with chaotic search, we developed a general learning method, CSBP, for SNN learning, which is similar to but different from CBP for MLP. In particular, it is not a trivial extension of CBP due to spiking signals (temporal code). From a neuroscience viewpoint, the dynamics of an SNN is similar to that of a biological brain (neural network), and thus the chaotic learning process of an SNN is brain-like learning in terms of temporal code and functional similarity. Mathematically, we can prove that the process of CSBP generates Marotto chaos initially, then undergoes various bifurcations, and eventually converges to a fixed point or solution (Note S2), which is consistent with the behavior of animal brains. As we expected, CSBP showed superior performance in the training or learning of SNNs. Specifically, CSBP outperforms existing SOTA methods on large-scale static data sets, such as ImageNet and CIFAR100, and on neuromorphic data sets, such as DVS-CIFAR10 and DVS-Gesture, with much higher accuracy. It is worth noting that, if we replace the chaos introduced by CSBP with the chaos generated by a chaotic map, such as a logistic map, the learning efficiency of SNNs will be drastically reduced (Fig. S12 and Note S12), which demonstrates the importance of biological plausibility in CSBP.

One important feature of chaos is that its long-term dynamics is almost independent of an initial value, significantly different from gradient dynamics, which depends on its initial value. Hence, CSBP is a global dynamic expected to be robust on the initial values and parameter selection—a notorious problem for traditional gradient-based BP methods. Another important feature of chaos is that chaotic dynamics is usually in fractal space, which is significantly smaller than the original state space in terms of volume, thereby improving the search efficiency. Therefore, there are two reasons for the superior performance of CSBP. First, the chaotic dynamics in CSBP is biologically plausible and can facilitate global search in the attractor region by its ergodicity. Thus, CSBP can achieve more efficient global optimization and generalization performance in SNNs than gradient-based and non-biological chaotic dynamics, such as chaotic learning rate and logistic mapping. In other words, the significant features of chaotic dynamics are chaotic itinerary (state transitivity or global dynamics) and fractal space (efficient search) in the attractor, which makes CSBP less dependent on the initial values (robust) and offers highly efficient search in contrast to the gradient dynamics (dependent on initial values or local dynamics, and non-efficient search). Second, due to the presence of the SG function in the existing methods, neurons remain activated or inhibited when the membrane potential exceeds a certain range, thus making it impossible for the network weights to be learned using error-based BP, which eventually leads to network degradation (Note S7 and Fig. S9). In contrast, the chaotic dynamics in CSBP enables the membrane potential to jump out of this region due to its pseudo-random nature.

Notice that, during initial training with CSBP, the output of the neuron ${h}_{ij}$ tends to a given value, ${I}_0 = 0.65$ (interior point), due to the form of cross-entropy of chaotic loss. This is fundamentally different from the BP-based method, which usually makes ${h}_{ij}$ to tend to be 0 or 1 (boundary points). From the optimization viewpoint, the relationship between CSBP and BP-based methods is similar to that between the interior point method and the simplex method (e.g. for linear optimization problems).

Although the forms of negative feedback of TCNN and CSBP are the same, the principles are different. This difference means that TCNN remains essentially a Hopfield network model [31,51], while CSBP becomes a general algorithm for training SNNs. Moreover, recent studies show that stochastic gradient descent (SGD) can make the training more effective in finding flat minima [52,53]. Our CSBP method has three features compared with SGD. Firstly, SGD randomly selects a subset of samples from the training set as input to the network during each iteration, while the chaos in CSBP is not random, but deterministic dynamics. Existing research has shown that the pseudo-randomness of chaos possesses a stronger exploration capability, e.g. chaotic itinerancy and global search ability in phase space. Secondly, SGD introduces external random noise to the neural network, independently of the network model. In contrast, CSBP utilizes the outputs of neurons as feedback, and its pseudo-randomness emerges internally from the network model. Thirdly, SGD was originally designed to address the issue of insufficient memory to input all samples at once. Although its convergence and generalization properties have been partially proven, it lacks a reasonable explanation in itself. In contrast, CSBP is designed to mimic chaotic dynamics in the brain, thus possessing biological plausibility. It is important to emphasize that CSBP and SGD are not mutually exclusive. As shown in Fig. S10 and Note S10, CSBP can be extended to the XOR classification task presented in Fig. 2. In fact, in this study, CSBP is combined with SGD and its variants (e.g. Adam) on the four benchmark data sets.

To the best of our knowledge, this study is the first to introduce biologically plausible chaotic dynamics to SNN direct training and opens a new approach in this area. We expect this work to have a broad positive impact on the community of SNNs or brain-inspired learning, with the following major features: (i) CSBP intrinsically introduces chaotic dynamics inspired by a real brain (see Note S3 for a thorough comparison of the CSBP algorithm with current experimental findings); (ii) CSBP theoretically guarantees its global search capability and convergence; (iii) CSBP can be also used as a plug-in unit to any existing method by adding only the last term of Equation (5), which in turn improves the performance, with no modification of the parameters of the existing method; (iv) CSBP is robust in terms of both initial conditions and hyperparameters; and (v) CSBP outperformed current SOTA methods on various data sets.

There is still room for improvement in CSBP. In our experiments, the chaotic loss can be introduced to the whole network or only to specific layers or neurons. We considered the computational cost and memory limitations in practical applications and added the chaotic term only in certain layers. However, it is unclear whether this is the best way to introduce chaos. Moreover, the two additional hyperparameters in CSBP, ${z}^0$and${\mathrm{\ }}\beta $, are currently set empirically. Although CSBP is robust to hyperparameters, it is necessary to set them adaptively, which can both extend the applicable scenarios and reduce the requirement on a priori knowledge. These issues will be addressed in our future work.

## METHODS

Detailed methods are given in the online Supplementary data.

## DATA AND CODE AVAILABILITY

The source code used in this work is available at https://github.com/Wangzj000/CSBP.

## Supplementary Material

nwae037_Supplemental_File
