# Supplementary material for: Brain-inspired chaotic spiking backpropagation
Source: Natl Sci Rev. 2024 Jan 30;11(6):nwae037. doi: 10.1093/nsr/nwae037 (PMC11067972; doi:10.1093/nsr/nwae037)
Supplement: nwae037_Supplemental_File [file nwae037_supplemental_file.pdf]

Supplementary Information

for

Brain-inspired Chaotic Spiking Backpropagation

Zijian Wang[1,#], Peng Tao[1,#], Luonan Chen[1,2,3,4,*]

[1] *Key Laboratory of Systems Health Science of Zhejiang Province, School of Life Science, Hangzhou Institute for Advanced Study, Hangzhou 310024; University of Chinese Academy of Sciences, China*

[2] *Key Laboratory of Systems Biology, Shanghai Institute of Biochemistry and Cell Biology, Center for Excellence in Molecular Cell Science, Chinese Academy of Sciences, Shanghai 200031, China.*

[3] *Guangdong Institute of Intelligence Science and Technology, Hengqin, Zhuhai, Guangdong 519031, China.*

[4] *Pazhou Laboratory (Huangpu), Guangzhou, Guangdong 510555, China.*

* To whom correspondence may be addressed: Luonan Chen: lnchen@sibs.ac.cn

[#] These authors contributed equally to this work

Methods

Pseudo-code of CSBP

The training or learning process of CSBP for minibatch gradient descent (other optimizers are similar) is shown in **Algorithm 1**. As a plug-and-play method, CSBP differs only in chaotic loss with an annealing variable from the BP-based baseline method. Clearly, **Algorithm 1** becomes the original BP method or its variant when removing loss_{chaos} term. Thus, it is easy to implement, e.g., as a plug-in unit to any BP-based method to improve its performance in terms of accuracy and robustness. In other words, CSBP is a general framework for a class of algorithms that can be CS-SGD, CS-Adam, and so on.

Algorithm 1. CSBP for mini-batch gradient descent

Input: training set $D = (X, Y)$, SNN model, learning rate η, maximal training epoch M, total training iteration in one epoch I, loss function L, initial chaotic intensity z^0, annealing constant β.

Randomly initialize all the weights w_{ijk} in the SNN model
for $m = 1, \dots, M$ **do**
    for $i = 1, \dots, I$ **do**
        Get the training data X^i and corresponding label Y^i
        Compute the output $\hat{Y}^i$ of the SNN model on i th batch
        Compute the target loss: $\text{loss}_{bp} = L(\hat{Y}^i, Y^i)$
        Compute the chaotic loss, loss_{chaos}, by Eq. (2)
        Update w_{ijk}: $w_{ijk} \leftarrow w_{ijk} - \eta \frac{(\partial \text{loss}_{chaos} + \partial \text{loss}_{bp})}{\partial w_{ijk}}$
        if annealing every training batch **then**
            $z^0 = z^0 \times \beta$
        End
    if annealing every epoch **then**
        $z^0 = z^0 \times \beta$
    End

Output: trained SNN model with w_{ijk}

Another implementation for CSBP

We have derived the original form of CSBP in Eq. (4), however, since $O_{i-1,k}^{t} = f\left(V_{i-1,k}^{t} - V_{th}\right)$, the gradient of $f(\cdot)$ is incontiguous, which may cause problems in optimizing the parameters of large-scale SNNs. Thus, we propose a variant of CSBP that has the same form of chaotic loss as Eq. (2) but with a different definition of h_{ij}, namely

$$h_{ij} = \text{sigmoid}\left(V_{ij}^{T}\right). \tag{8}$$

The derivation is given in Supplementary Note 1. This implementation can bring analogous chaotic dynamics for the weight in SNNs like the preceding implementation. A numerical experiment is shown in Supplementary Fig. 1. In our experiments on four benchmarks, the chaotic loss is introduced in the form of the second implementation. Notably, since current SNN models treat the spiking rate of the previous layer as an input and the spiking rate of the current layer as an output and run a LIF model in a time window, CSBP merely needs the memory of the spiking rate on one layer and releases the memory when backward to another layer, which brings no extra memory consuming. Moreover, for the variant of CSBP (Eq. (8)), it requires only the membrane potential at the last time point to compute the chaotic loss. Therefore, CSBP matches the limited memory capacity of real biological systems.

Datasets and pre-processing

Four benchmark datasets, CIFAR100, ImageNet, DVS-CIFAR10 and DVS-Gesture, were used in our experiments. DVS-CIFAR10 is a mainstream neuromorphic dataset or event-stream dataset, with 10000 128×128 images for 10 classes, which is suitable for testing the performance of SNNs. We utilized the same data pre-processing method in [25]. DVS-Gesture is collected by 29 individuals with real-time events, which contains 1342 instances of 11 classes. CIFAR100 is one of the most used datasets in the image classification field, which contains 60000 32×32 color images in 100 classes

with 50000 images in the training set and 10000 images in the test set. ImageNet is one of the most challenging large-scale datasets. It provides 1280 images per class for 1000 classes in total. Data augmentation was applied in our experiments. We cropped the images to the size of 224×224 with normalization.

Hyperparameters and training

When applying CSBP to a given baseline method (i.e., SEW and TET), the hyperparameters of the corresponding baseline method were directly used, except for two hyperparameters of chaotic loss. Until now, there is no effective scheme to automatically regulate these two hyperparameters, so we need to set the hyperparameters based on an empirical scheme rather than a self-regulation scheme (see Supplementary Note 8 for the empirical scheme). Specifically, for CIFAR100, the chaotic loss was introduced into the last linear layer of Spiking ResNet-19 with chaotic intensity annealing every epoch ($z^0 = 15, \beta = 0.997$). For DVS-CIFAR10, the chaotic loss was introduced into the two down-sampling convolutional layers of Wide-7B-Net with chaotic intensity annealing every epoch. When T = 4 and 8, z^0 was set to 10 and 25, and β was set to 0.99 and 0.995, respectively. The chaotic intensity was annealed every epoch. For DVS-Gesture, the chaotic loss was introduced into the first down-sampling convolutional layer in 7B-Net with chaotic intensity annealing every epoch ($z^0 = 15, \beta = 0.95$). For ImageNet, the chaotic loss was introduced into the first down-sampling convolutional layer and the first residual block of SEW ResNet-18 and SEW ResNet-34, respectively. The chaotic intensity was annealed every training batch and $z^0 = 20, \beta = 0.999997$ for both cases. Notably, existing works [36, 54] (see Fig. 4) show that the values of hyperparameters are usually valid in a wide interval, so setting them manually is not a difficult task, however we will further investigate the self-tuning scheme in depth in our future work. Moreover, Zhang et al. [55] show that hyperparameters can also induce chaotic dynamics in SNN, e.g., k_τ. But CSBP can generate chaotic dynamics regardless of them (Supplementary Note 4 and

Supplementary Fig. 2).

References (continue)

54. Liu B, Wang L and Jin Y-H *et al.* Improved particle swarm optimization combined with chaos. *Chaos Solitons Fractals*. 2005; **25**: 1261-1271.

55. Zhang S-Q, Zhang Z-Y and Zhou Z-H. Bifurcation spiking neural network. *J Mach Learn Res*. 2021; **22**: 11459-11479.

Supplementary Note 1. The second implementation of CSBP

In the main text, we provide the first implementation of CSBP. In this section, we describe its second implementation. Here we define chaotic loss as $\text{loss}_{\text{chaos}} = -\sum_{i=1}^{l}\sum_{j=1}^{M_i} z_{ij}\left[I_0 \ln h_{ij} + (1-I_0)\ln\left(1-h_{ij}\right)\right]$, where $h_{ij} = \text{sigmoid}\,(V_{ij}^T)$. The gradient of w_{ijk} is $\frac{\partial \text{loss}_{csbp}}{\partial w_{ijk}} = -z_{ij}(I_0 - h_i)\frac{\partial V_{ij}^T}{\partial w_{ijk}}$. We can derive that the expression of $\frac{\partial V_{ij}^T}{\partial w_{ijk}}$ is recursive, which is $\frac{\partial V_{ij}^T}{\partial w_{ijk}} = [1 - k_\tau V_{ij}^{T-1} f'(V_{ij}^{T-1} - V_{th})]O_{i-1,k}^T + k_\tau(1 - O_{ij}^{T-1})\frac{\partial V_{ij}^{T-1}}{\partial w_{ijk}}$. The updating formula of w_{ijk} for all (i,j,k) can be written as:

$$\begin{aligned} w_{ijk} &\leftarrow w_{ijk} - \eta\frac{\partial \text{loss}_{csbp}}{\partial w_{ijk}} \\ &= w_{ijk} - \eta\frac{\partial \text{loss}_{bp}}{\partial w_{ijk}} - \eta\frac{\partial \text{loss}_{chaos}}{\partial w_{ijk}} \qquad , \\ &= w_{ijk} - \eta\frac{\partial \text{loss}_{bp}}{\partial w_{ijk}} + \eta z_{ij}(I_0 - h_i)\frac{\partial V_{ij}^T}{\partial w_{ijk}} \end{aligned} \tag{S1}$$

In practice, V_{ij}^T may be relatively large or even fall into the saturation region, so we use the steepness parameter for the sigmoid function, which is $h_{ij} = \text{sigmoid}\left(\frac{V_{ij}^T}{\epsilon}\right) = \frac{1}{1+e^{-V_{ij}^T/\epsilon}}$. Here ϵ is the steepness parameter, and we set $\epsilon = 10$ for SEW ResNet-34 and SEW ResNet-50 on ImageNet. A numerical experiment on a single spiking neuron is shown in Supplementary Fig. 1. The initial chaotic intensity z^0 was set to 15 and the annealing constant β was set to 0.9997. Similarly to Fig. 2, the input of the neuron is 0 and the target output is 1. Here we trained the neuron for 4000 epochs.

When V_{ij}^T is too large or small, h_{ij} will approach 0 or 1, and then $\text{loss}_{\text{chaos}}$ will approach infinity. Here we clip the range of the sigmoid function, which is $h_{ij} = \text{sigmoid}(9.9), \mathrm{V}_{\mathrm{ij}}^{\mathrm{T}} > 9.9$ and $h_{ij} = \text{sigmoid}(-10), \mathrm{V}_{\mathrm{ij}}^{\mathrm{T}} < -10$.

Supplementary Note 2. Marotto chaos of CSBP

In this section, we will mathematically show that CSBP can generate chaotic dynamics when the self-negative feedback or temperature parameter z_{ij} is sufficiently strong. To make the proof clearer, we use the simplified CSBP form of Eq. (5) or (S1) with m as the updating iteration as follows:

$$\begin{aligned} w_{ijk}^{m+1} &= w_{ijk}^{m} - \eta \frac{\partial \mathrm{loss}_{bp}}{\partial w_{ijk}} + z_{ij}\big(I_0 - h_{ij}\big) \\ &= w_{ijk}^{m} + a_{ijk}(W^m) + z\left(I_0 - h_{ij}(W^m)\right), \end{aligned} \tag{S2}$$

where $a_{ijk}(W^m) = -\eta \frac{\partial \mathrm{loss}_{bp}}{\partial w_{ijk}}$, $z_{ij} \leftarrow \eta z_{ij} \frac{1}{T} \sum_{t=1}^{T} O_{i-1,k}^{t}$ for Eq. (5) or $\eta z_{ij} \frac{\partial V_{ij}^{T}}{\partial w_{ijk}}$ for Eq. (S1), and further we adopt a common positive scalar z ← z_{ij} for all neurons in Eq. (S2) rather than separative z_{ij} for each neuron, which is a temperature parameter in the annealing process. The output/activation function of Eq. (5) is rewritten as

$$\begin{aligned} h_{ij}(W^m) &= \frac{1}{1 + \exp\left(-\frac{1}{T}\sum_{t=1}^{T}\sum_{k=1}^{M_{i-1}} w_{ijk}^{m} O_{i-1,k}^{t}\right)} \\ &= \frac{1}{1 + \exp\left(-\sum_{k=1}^{M_{i-1}} \frac{w_{ijk}^{m}}{M_{i-1}\varepsilon_{ik}}\right)}. \end{aligned} \tag{S3}$$

where ε_{ik}=$T/[M_{i-1}\sum_{t=1}^{T} O_{i-1,k}^{t}]$. All other variables or parameters are defined in Eq. (5) or Eq. (S1). W^m is a vector of w_{ijk}^{m} for all (i,j,k) with i,j,k=1,2,…; m is the iteration number with m=0,1,... The vector form of Eq. (S2) can be written as

$$W^{m+1} = G(W^m) \tag{S4}$$

where W^m is a vector of w_{ijk}^{m} for all (i,j,k), and G is a vector function of W^m for all (i,j,k). Each element $g_{ijk}(W^m)$ of $G(W^{m+1})$ is the right-hand side of Eq. (S2). Then, we first prove that there exists a fixed point for W^m of Eq. (S2) or (S4) when z is sufficiently large.

Theorem 1: Assume $1 > I_0 > 0$, and z is sufficiently large. Then W of Eq. (S2) or (S4) has a unique fixed point which is a repeller $\overline{W}$ with each element as

$$\bar{w}_{ijk} = \varepsilon_{ik}\ln\frac{I_0}{1-I_0} + \mathcal{O}\left(\frac{1}{z}\right) \quad \text{for } i,j,k = 1,2,...,$$

where $\mathcal{O}\left(\frac{1}{z}\right)$ means a term with the order of $\frac{1}{z}$ when z is sufficiently large.

Proof of Theorem 1: From Eq. (S2), a fixed point $\bar{W}$ with $W^{m+1} = W^m$ satisfies

$$a_{ijk}(\bar{W}) + z\left(I_0 - h_{ij}(\bar{W})\right) = 0, \text{ i.e., } h_{ij}(\bar{W}) = I_0 + a_{ijk}(\bar{W})/\text{z}.$$

Thus, it is easy to show $\bar{w}_{ijk} = \varepsilon_{ik}\ln\frac{I_0}{1-I_0} + \mathcal{O}(\frac{1}{z})$ satisfying both the necessity and sufficiency of a unique fixed point for Eq. (S2). In other words, each element of this fixed point $\bar{W}$ is in the neighbor of $w_{ijk}^{\infty} = \varepsilon_{ik}\ln\frac{I_0}{1-I_0}$ for *i,j,k*=1,2,…, when *z* is sufficiently large. Furthermore, it can easily check all eigenvalues of the Jacobian matrix $\frac{dG(W)}{dW}|_{\bar{W}}$ for W^m of Eq. (S2) or (S4) at this fixed point $\bar{W}$ are more than 1 in terms of the norm, i.e. $\bar{W}$ is a repeller. The detail proof procedure is similar to the Appendix A and B of [1] or can also refer to those in [2, 3].

Next, we will show this unique fixed point $\bar{W}$ is actually a snap-back repeller, which generates Marotto chaos [1, 2], when *z* is sufficiently large.

Theorem 2: Assume $1 > I_0 > 0$ with $I_0 \neq 0.5$, and *z* is sufficiently large. Then $\bar{W}$ of Eq. (S2) or (S4) is a snap-back repeller, or Eq. (S2) or (S4) has a transversal homoclinic orbit which generates chaotic dynamics in the sense of Marotto. In particular, the following point $\bar{W}^0$ with each element as

$$\bar{w}_{ijk}^0 = \varepsilon_{ik}\ln\frac{2I_0-1}{2-2I_0} + \mathcal{O}(\frac{1}{z}) \text{ for } I_0 > 0.5, \text{ or, } \bar{w}_{ijk}^0 = \varepsilon_{ik}\ln\frac{2I_0}{1-2I_0} + \mathcal{O}(\frac{1}{z}) \text{ for } I_0 < 0.5$$

for *i,j,k*=1,2,…, is on this homoclinic orbit. Here $\mathcal{O}\left(\frac{1}{z}\right)$ means a term with the order of $\frac{1}{z}$, when *z* is sufficiently large.

Proof of Theorem 2:

Letting W^0 with each element as

$$w_{ijk}^{0}=\varepsilon_{ik}\ln\frac{2I_0-1}{2-2I_0} \quad \text{or} \quad \varepsilon_{ik}\ln\frac{2I_0}{1-2I_0}$$

be the initial state/point of Eq. (S2) or (S4), we have the next point $W^1=G(W^0)$ as

$$\begin{aligned} w_{ijk}^{1} &= w_{ijk}^{0}+a_{ijk}(W^0)+z\left(I_0-h_{ij}(W^0)\right) \\ &= \mathcal{O}(1)+z(1-I_0) \text{ or } \mathcal{O}(1)-zI_0 \end{aligned} \tag{S5}$$

due to $h_{ij}(W^0)$=2I_0-1 for $I_0>0.5$, or $h_{ij}(W^0)$=2I_0 for $I_0<0.5$. Note that $I_0\neq 0.5$ and $w_{ijk}^{0}\neq w_{ijk}^{\infty}$. Then, the next iteration for $W^2=G(W^1)=G\big(G(W^0)\big)$ is

$$\begin{aligned} w_{ijk}^{2} &= w_{ijk}^{1}+a_{ijk}(W^1)+z\left(I_0-h_{ij}(W^1)\right) \\ &= \mathcal{O}(1)+z(1-I_0)+z\big(I_0-1+\mathcal{O}(e^{-a_0 z})\big) \text{ or } \mathcal{O}(1)-zI_0+z\big(I_0+\mathcal{O}(e^{-b_0 z})\big) \\ &= \mathcal{O}(1) \end{aligned} \tag{S6}$$

where $a_0=(1-I_0)\sum_{k=1}^{M_{i-1}}\frac{1}{M_{i-1}\varepsilon_{ik}}>0$; $b_0=I_0\sum_{k=1}^{M_{i-1}}\frac{1}{M_{i-1}\varepsilon_{ik}}>0$.

Hence, from the derivations above, we can use Urabe's proposition [4] to prove this theorem. We construct

$$Q(W;z)=\frac{1}{z}\left[G\big(G(W)\big)-\bar{W}\right],$$

then, from Eqs. (S5)-(S6), we have

$$Q(W^0;z)=\frac{1}{z}\left[G\big(G(W^0)\big)-\bar{W}\right]=\frac{[\mathcal{O}(1)]}{z}.$$

Hence,

$$\lim_{z\to\infty}Q(W^0;z)=[0],$$

where $[\mathcal{O}(1)]$ is a vector with all $\mathcal{O}(1)$ elements, and [0] is a vector with all 0 elements.

We can show the all eigenvalues of the Jacobian matrix $\frac{dQ(W;z)}{dW}|_{W^0}$ of $Q(W;z)$ at W^0 are not zero when z is sufficiently large. Thus, according to Urabe's proposition [1, 2, 4], $Q(W;z)=0$ has a unique solution W^{0*} near W^0, i.e. when z is sufficiently large,

$$G(G(W^{0*}))=\bar{W}.$$

Furthermore, all eigenvalues for the Jacobian matrix $\frac{dG(W)}{dW}|_{W^{0*}}$ of G(W) at W^{0*} exceed unity in the norm when z is sufficiently large, and thus the intersection of the global stable and unstable sets at the homoclinic point W^{0*} is transversal. Note the preimages $G^{-m}(W^{0*})$ in an appropriate neighbor of the repeller $\bar{W}$ converge to $\bar{W}$ when z is

sufficiently large. Thus $\overline{W}$ of Eq. (S1) is a snap-back repeller [1, 2, 3], which generates chaos. The detailed procedure of the proof is similar to those in [1, 2, 3].

Marotto chaos is a topological chaos in a high-dimensional system R^n, which can be viewed as a generalized Li-Yorke chaos in a one-dimensional system R [5, 6]. Marotto chaos means that the system has any p-periodic points, and at the same time has a scrambled set or an uncountable set containing no periodic points with topological transitivity. In this work, we explore both topological and numerical chaos for SNNs. For numerical chaos, we explore the ergodicity or sensitivity of initial values as well as the fractal space of the attractor so as to make the algorithm robust and efficient. Specifically, one important feature of chaos is that long-term dynamics is almost independent of its initial value, significantly different from the gradient dynamics that is dependent on its initial value. Thus, CSBP is a global dynamics and is expected to be robust on the initial values and parameters selection, which are actually a notorious problem for traditional gradient-based BP methods. Another important feature of chaos is that the dynamics is on the fractal space, which is significantly smaller than the original state space in terms of volume, thus improving the searching efficiency.

Supplementary Note 3. Biological plausibility of the CSBP algorithm

In order to better articulate the biological plausibility of the CSBP algorithm, we dug deeper into the relevant experimental and theoretical models. Hodgkin and Huxley[8] proposed a set of differential equations (the HH model) to quantitatively describe the generation and conduction of impulses on neurons, based on their study of the squid giant axon. However, the HH model is too complex for practical applications. Therefore, people simplified the HH model and obtained some more feasible models, such as the FitzHugh–Nagumo model[9, 10]. Nagumo and Sato[11] further simplified the model and reduced the computational cost while preserving the qualitative description of neuronal dynamics. After that, several experiments observed chaotic dynamics in the learning process of the brain. For example, Skarda and Freeman's experiment in 1987 showed that chaotic dynamics of brain neurons are indispensable for rabbits to acquire new olfactory patterns[12]. Similarly, Matsumoto et al. found that the nervous system of an octopus perceives external information using chaotic dynamics[13]. To model these observed chaotic dynamics in the real brain, Aihara introduced the idea of chaotic neural network[14] based on the Nagumo–Sato model and successfully incorporated biologically meaningful chaos into neural networks. Later, Chen and Aihara developed transient chaotic neural networks (TCNN) with chaotic simulated annealing (CSA), which were applied to combinatorial optimization problems. Since the chaos introduced by our CSBP algorithm is consistent with that in TCNN, which is based on a simplified HH model (Nagumo–Sato model), we consider the CSBP algorithm biologically reasonable. It is important to note that what we call biological plausibility is qualitative rather than quantitative, as it derives from existing theoretical models describing the dynamics of chaos in the brain, and there are some approximations of these theoretical models. In addition, there are also a number of studies to show that learning and recognition in a brain explore the chaotic dynamics[15, 16].

Supplementary Note 4. CSBP can generate chaotic dynamics on different SNN hyperparameters

Previous studies have confirmed that LIF SNN models can generate bifurcations in the evolution of neuro-dynamics under certain LIF hyper-parameters[7]. However, CSBP introduces transient chaotic dynamics in SNN training. It is still worth investigating on the possible connection between the internal bifurcations in LIF neurons and the chaotic dynamics of CSBP learning.We conducted experiments on the existence of chaotic dynamics on different SNN hyper-parameters, $\boldsymbol{k_{\tau}}$. On a single LIF neuron, we set different $\boldsymbol{k_{\tau} = 0.2, 0.4, 0.6, 0.8, 1}$ and found that CSBP indeed generates chaotic dynamics regardless of LIF hyperparameters (Supplementary Fig. 2). The initial chaotic intensity $\boldsymbol{z^{0}}$ was set to 20 and the annealing constant $\boldsymbol{\beta}$ was set to 0.9995.

Supplementary Note 5. Different $\boldsymbol{I_0}$ of CSBP on a single neuron

As regards to I_0, it was usually set to a default value of 0.65, thus in CSBP I_0 was also set to 0.65 for simplicity. But it should be noted that theoretically CSBP can always generate chaos (e.g. Marotto chaos) when $I_0 \neq 0.5$. To illustrate the effect of I_0, we investigate a single neuron with different $I_0 = 0.2, 0.4, 0.65, 0.8$. As shown in Supplementary Fig. 3, regardless of the value of I_0, CSBP always produces chaotic dynamics. The initial chaotic intensity z^0 was set to 20 and the annealing constant β was set to 0.9995.

Supplementary Note 6. Apply CSBP to a small SNN for regression task

We implemented CSBP on a small SNN with the structure of [3, 10, 5] for a regression task. The input was uniform sampled from the range of [-0.5, 0.5] and the target was

0.3 for all dimensions. We can observe similar chaotic dynamics and global search capability in the regression task (Supplementary Fig. 4).

Supplementary Note 7. CSBP can avoid network degradation

We used the single spiking neuron model. The initial value of w was set to 2 and the initial value of b was set to 1.6. MSE loss was used. The surrogate function was $f(V) = \text{sign}(|V - V_{th}| < 0.5)$. The membrane threshold was set to 1. Thus, $f(V) \neq 0$ when $0.5 < V < 1.5$, but the initial value of the single neuron membrane potential was 3.6. The neuron is "dead" and cannot be trained with BP (Supplementary Fig. 9). In contrast, when training with CSBP, we can see the chaotic dynamics of the neuron and the neuron can be trained effectively.

Supplementary Note 8. Empirical scheme for setting hyperparameters

In our study, the two hyperparameters (z^0 and β) of the CSBP are set empirically rather than using self-regulation. This is because, in order for the CSBP to achieve optimal performance, z^0 needs to be large enough to ensure that the globally optimal attractor region can be sampled, and β needs to be sufficiently large to ensure that the annealing rate is slow enough. However, too large z^0 and β will significantly increase the inefficient computation and thus degrade the performance of the CSBP. Until now, there is no effective scheme to automatically regulate these two hyper-parameters, so for each dataset, we need to set the hyper-parameters based on some priori knowledge. Using an average on the number of neurons used for computing chaotic loss is recommended. To simplify the hyperparameter setting, according to the main text, we only need to set two hyperparameters: z^0 and β. Supplementary Table 1 shows the setting in our experiments. It can be seen that both the two annealing strategies may work (i.e., annealing per batch or epoch). Using an average on the number of neurons used for computing chaotic loss is recommended. Specifically, , the initial chaotic intensity (z^0) should be set in a reasonable interval: $[10, 25]$. The annealing constant (β) should satisfy $0 < \beta < 1$, but it is better to set $\beta > 0.9$ to ensure the chaotic intensity while training to fall slowly not too fast, which can keep the chaotic dynamics in the initial training process. However, to avoid slow convergence, β should not be too large. The chaotic loss should be less than the BP loss in the last second half training process. Notably, when chaotic intensity anneals every epoch, β is closely related to the size of the dataset (e.g., β was set to be 0.999997 in ImageNet for the size of ImageNet training set is $1{,}281{,}167$).

Supplementary Note 9. The computation of Lyapunov exponent.

To calculate Lyapunov exponents, for the value of z at each epoch, we initialized a new network with the same hyper-parameters and trained it with the initial chaotic intensity $z^0 = z$ without annealing for 3000 epochs. The Lyapunov exponent of the weight was computed by nolds (https://pypi.org/project/nolds/) in Python, representing the Lyapunov exponent of the original network at one epoch. According to the original algorithm, the computation includes three steps: 1) reconstructing the dynamics in a finite-dimensional space; 2) obtaining the tangent maps of the reconstructed dynamics; 3) deducing the Lyapunov exponent from the tangent maps. Specifically, we denote the time series as $[x_1, x_2, \ldots, x_{N-1}]$ and embed it into a d_E-dimensional orbit at each step x_i, $\boldsymbol{X_i} = \left[x_i, x_{i+1}, x_{i+2}, \ldots\ x_{i+d_E-1}\right]$. For each $\boldsymbol{X_i}$, find a radius r_s and determine all $\boldsymbol{X_j}$ satisfying $\left|\boldsymbol{X_j} - \boldsymbol{X_i}\right| \leq r_s$ are $\boldsymbol{X_i}$'s neighbors. Find the matrix $\boldsymbol{T_i}\left(\boldsymbol{X_j} - \boldsymbol{X_i}\right) \cong \boldsymbol{X_{j+m}} - \boldsymbol{X_{i+m}}$ to describe the time evolution by linear least squares fit, where m is the step size setting to avoid undetermined values in $\boldsymbol{T_i}$. Starting with $i = 1$ and $\boldsymbol{Q_0}$, we successively decompose the matrix $\boldsymbol{T_i} \times \boldsymbol{Q_{i-1}}$ into the matrices $\boldsymbol{Q_i}$ and $\boldsymbol{R_i}$ by a QR decomposition. Finally, calculate the Lyapunov exponents from the mean of the logarithm of the diagonal elements of the matrices $\boldsymbol{R_i}$.

Supplementary Note 10. CSBP in the minibatch scheme

To validate the effectiveness of CSBP in the minibatch scheme, we generated 200 data points for the XOR task, as illustrated in Supplementary Fig. 10a (the red dots and blue stars represent the two classes). The settings were consistent with those described in Fig. 2. Each batch consisted of 32 randomly selected data points from the dataset. The chaotic intensity z and the annealing constant β were set to 20 and 0.9999, respectively, with annealing performed every epoch. The dynamic curves of w_{220} and loss_{csbp} are presented in Supplementary Figs. 10b and c. When employing the minibatch scheme, CSBP was capable of generating similar chaotic dynamics as depicted in Fig. 2.

Supplementary Note 11. The respective roles of loss_{chaos} and loss_{bp}.

The role of loss_{chaos} is to facilitate the exploration of a broader weight space, thereby increasing the chances of finding the global optimum. On the other hand, loss_{bp} represents the original objective loss for optimization, ensuring the eventual convergence of an SNN model. To provide a clearer understanding of their respective roles, we present the dynamics of a single-layer SNN at three different stages. In the first stage (first 3500 epochs), the magnitude of loss_{chaos} significantly exceeds that of loss_{bp}. During this phase, loss_{chaos} dominates the weight dynamics. As shown from the Supplementary Fig. 11d, the network explores a wide range in the weight space to search for potential global optimum. In the second stage (3500-8000 epochs), as the chaos intensity z anneals, loss_{chaos} gradually decreases to a level comparable to that of loss_{bp}. At this point, the weight dynamics exhibit rich fluctuation behaviors, allowing the network to conduct localized and in-depth explorations within the weight space (Supplementary Fig. 11e). In the third stage (final 2000 epochs), with further annealing of z, loss_{chaos} approaches 0. Consequently, loss_{bp} takes control of the weight dynamics, and CSBP degenerates into BP. Ultimately, the convergence of training is ensured through the BP gradients (Supplementary Fig. 11f). The learning process was conducted on a one-layer SNN with dimensions [10, 3]. Input data and target data were randomly generated from the range [-1, 1]. The simulation time T was set to 10.

Supplementary Note 12. Chaotic optimizers using Logistic map.

the ergodicity is an essential property of chaos when applied to optimization problems. However, not all chaos is suitable for training SNNs. To demonstrate this point, we chose a simple yet widely used logistic map to generate chaos. Without loss of generality, we denote the chaos term introduced in the weight update formula as $f(w_{ijk})$. Thus, we have $w_{ijk} \leftarrow w_{ijk} - \eta \frac{\partial \mathrm{loss}_{bp}}{\partial w_{ijk}} + f(w_{ijk})$. For CSBP, $f(w_{ijk}) = \eta z_{ij}(I_0 - h_{ij}) \frac{1}{T} \sum_{t=1}^{T} O_{i-1,k}^{t}$. If we use the logistic map, then $w_{ijk}^{t+1} = r w_{ijk}^{t}\left(1 - w_{ijk}^{t}\right)$, where $r \in [0, 4]$ is a hyperparameter controlling the chaos. When training with this chaos, the resulting dynamic trajectories are shown in Supplementary Figs. 12a and b for $r = 3.82$ and $r = 3.92$, respectively. Comparing these trajectories with the CSBP trajectories in Fig. 2, it is evident that the weight space explored using the logistic map is much smaller, leading to a significant degradation in the learning performance. One possible explanation is that the logistic map is directly introduced from external sources, resulting in high similarity for different weights even with strong chaos. In contrast, the chaos in CSBP is generated internally by the network, dynamically adapting to changes in neuron outputs, thereby exhibiting better adaptability. In conclusion, compared to external or general chaotic dynamics, the internal or intrinsic chaotic dynamics in the brain appear to be more efficient. However, the specific form of chaos in the brain remains an unclear question that we will further explore in our future work. The learning process was conducted on a one-layer SNN with dimensions [10, 3]. Input data and target data were randomly generated from the range [-1, 1]. The simulation time T was set to 8.

Supplementary Table 1. Hyperparameters of CSBP used in this work.

Dataset	T	max epoch	z^0	β	Annealing strategy
DVS-CIFAR10	4	64	10	0.99	every batch
	8		25	0.995	
DVS-Gesture	16	192	15	0.95	every epoch
CIFAR100	2	300	15	0.997	every epoch
	4				
	6				
ImageNet	4	320	20	0.999997	every batch

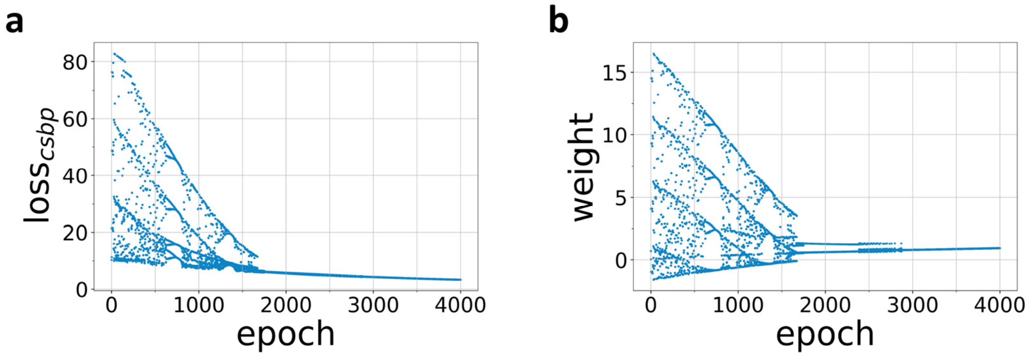

Supplementary Fig. 1. A toy example of a single spiking neuron with the second CSBP implement. **(a)** The CSBP loss curve of the single spiking neuron while training. **(b)** The dynamic curve of weight change of the single spiking neuron while training.

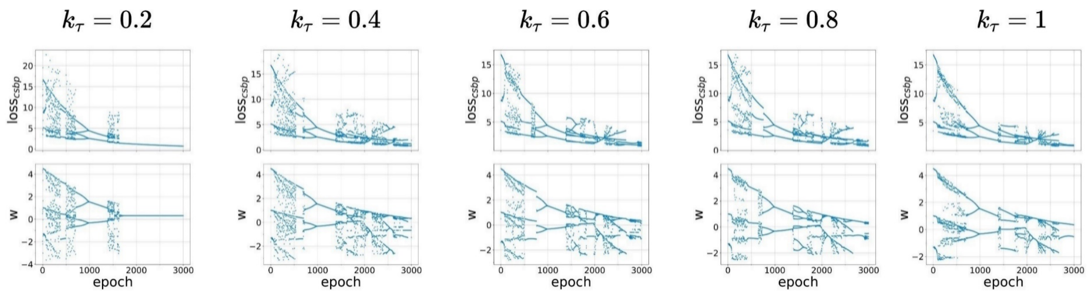

Supplementary Fig. 2. A toy example of a single spiking neuron with CSBP trained. The membrane constant $k_\tau = 0.2, 0.4, 0.6, 0.8, 1$ in each column.

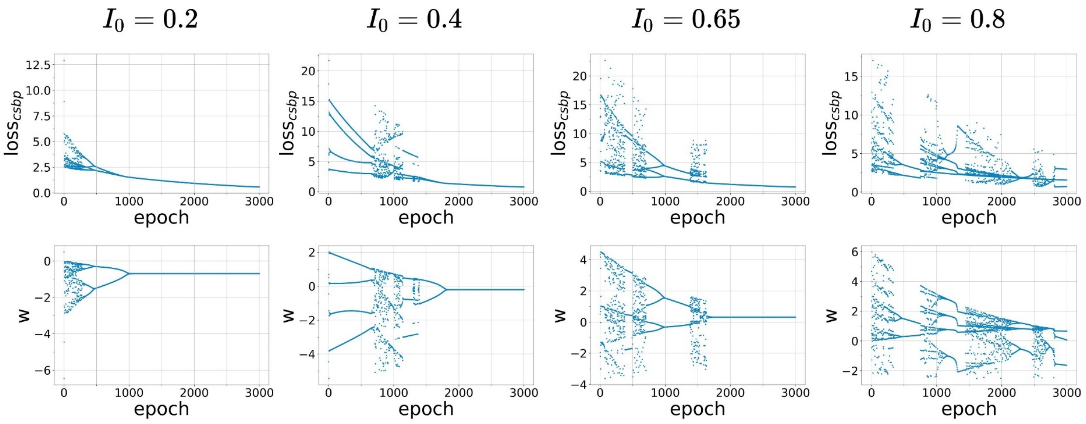

Supplementary Fig. 3. A toy example of a single spiking neuron with CSBP trained. The membrane constant $I_0 = 0.2, 0.4, 0.65, 0.8$ in each column.

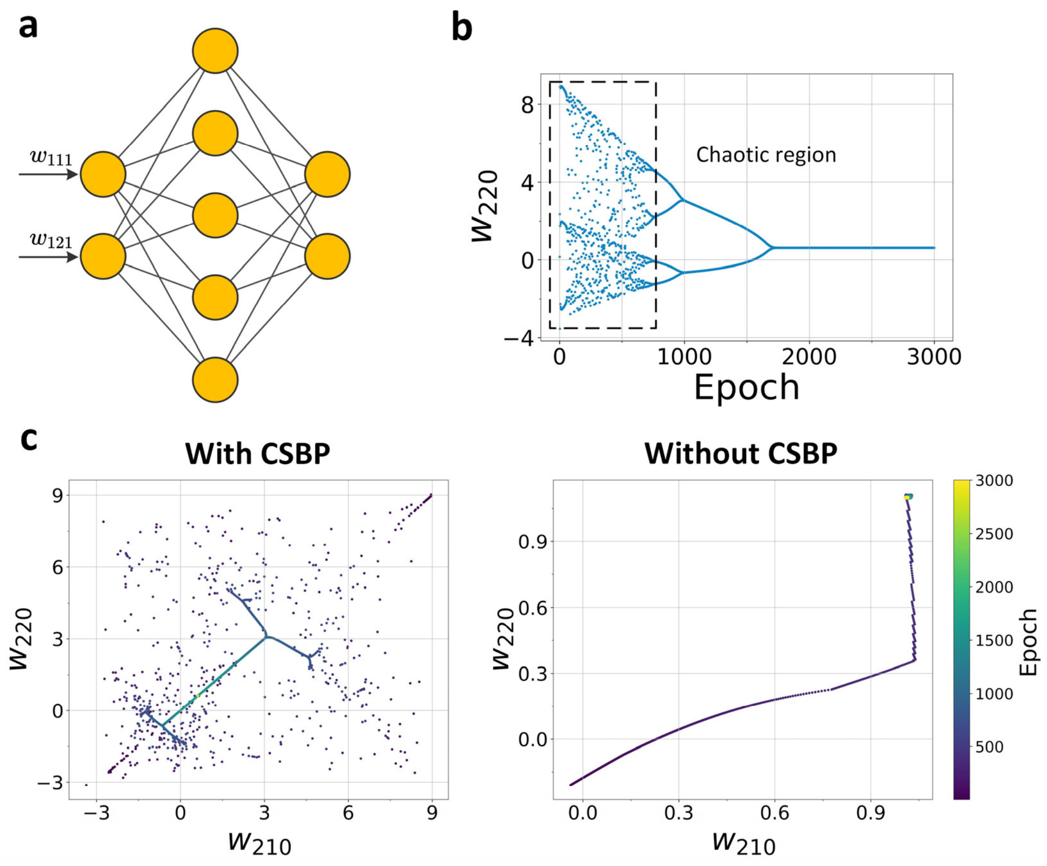

Supplementary Fig. 4. Global searching ability of CSBP on regression task. (a) An SNN was used for a regression task. The input was uniformly sampled from the range of [-0.5, 0.5] and the target was 0.3 for all dimensions. (b) With the chaotic strength decreasing, the weight w_{220} in the regression task exhibits chaotic dynamics and converges after searching. The dashed box shows a chaotic region when chaotic strength is sufficiently large. (c) The learning dynamic curves in the weight space (w_{220} and w_{122}, w_{220} and w_{210}) while training with CSBP and without CSBP in the regression task.

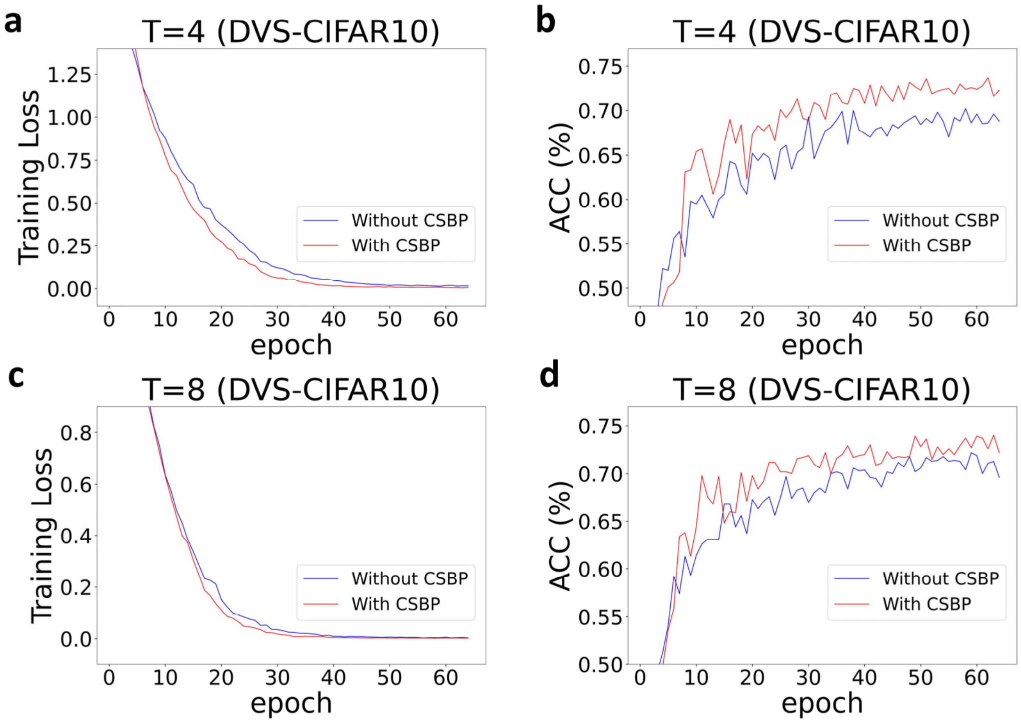

Supplementary Fig. 5. Comparison of CSBP and the baseline method without CSBP introduced on the DVS-CIFAR10 dataset. The red lines represent SNN training with CSBP and the blue lines represent SNN training without CSBP. **(a)** and **(b)** are the results of training loss and test accuracy with a simulation time of 4 respectively. **(c)** and **(d)** are the results of training loss and test accuracy with a simulation time of 8 respectively.

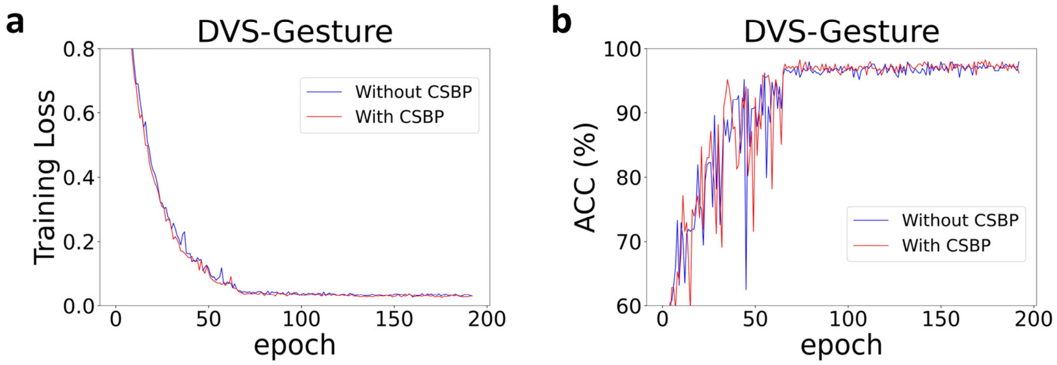

Supplementary Fig. 6. Comparison of CSBP and the baseline method without CSBP introduced on the DVS-Gesture dataset. The red lines represent SNN training with CSBP and the blue lines represent SNN training without CSBP. **(a)** and **(b)** are the results of training loss and test accuracy respectively.

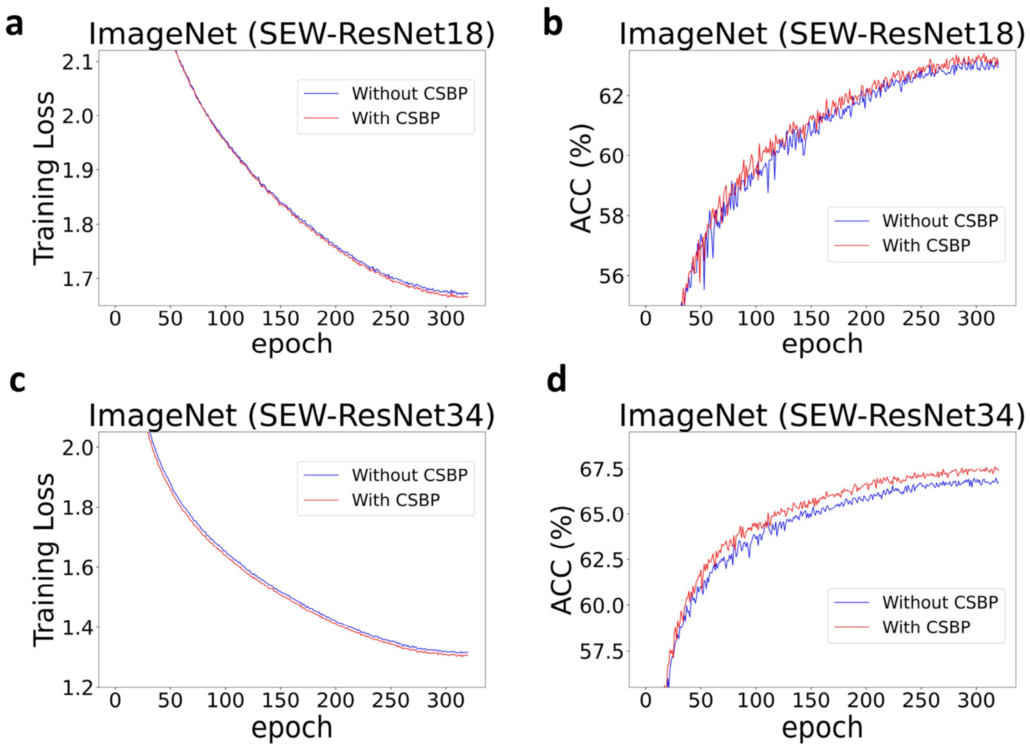

Supplementary Fig. 7. Comparison of CSBP and the baseline method without CSBP introduced on the ImageNet dataset. The red lines represent SNN training with CSBP and the blue lines represent SNN training without CSBP. **(a)** and **(b)** are the results of training loss and test accuracy on SEW-ResNet18 respectively. **(c)** and **(d)** are the results of training loss and test accuracy on SEW-ResNet34 respectively.

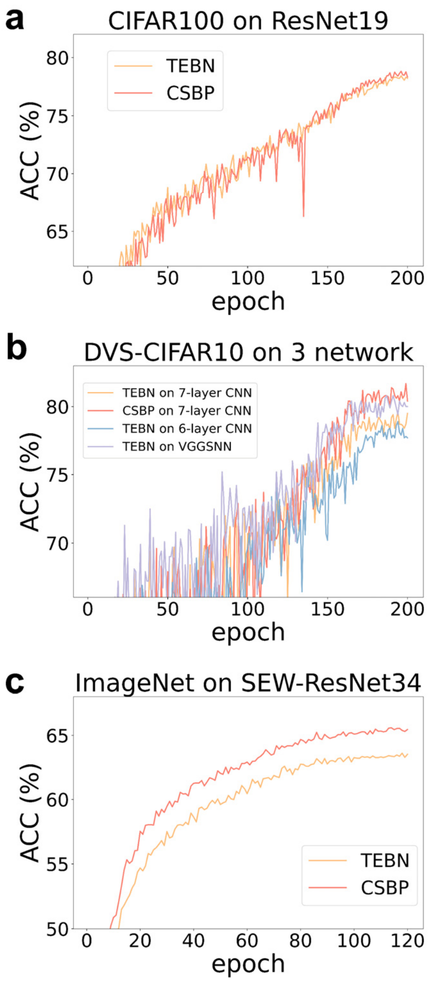

Supplementary Fig. 8. Comparing the performance of CSBP and TEBN.

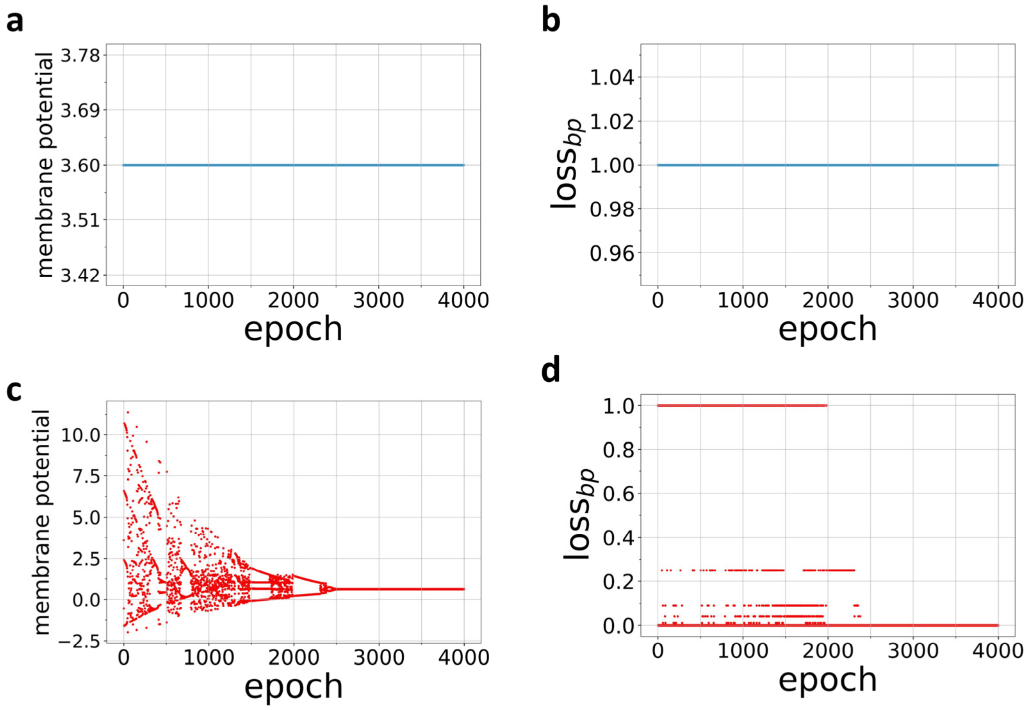

Supplementary Fig. 9. Comparison of CSBP and the method without CSBP introduced in a “dead” spiking neuron. **(a)** The membrane potential curve of BP. **(b)** The BP loss (MSE loss) curve of BP. **(c)** The membrane potential curve of CSBP. **(d)** The BP loss (MSE loss) curve of BP.

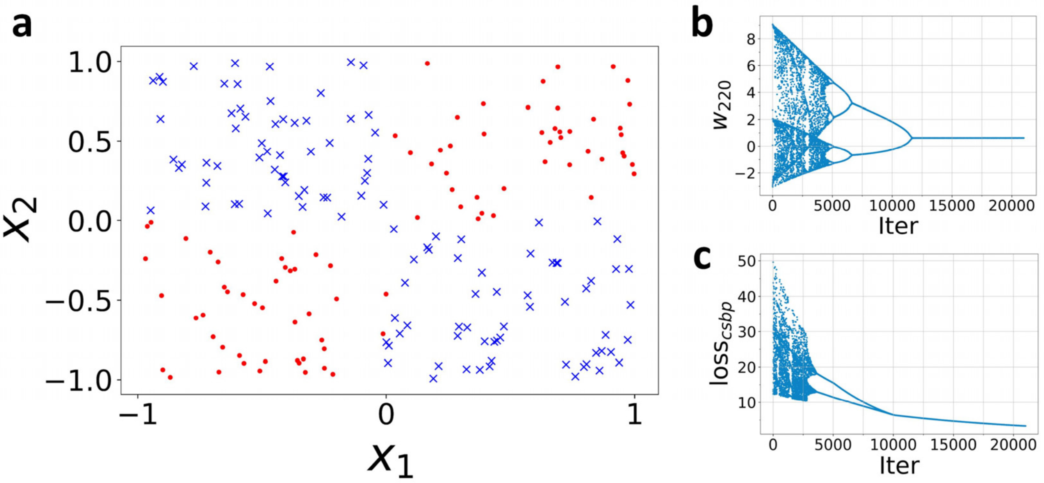

Supplementary Fig. 10. A toy XOR example combining CSBP with a minibatch scheme. (**a**) The 200 randomly generated data. (**b**) The iteration curve of weight w_{220}. (**c**) The iteration curve of loss_{csbp}.

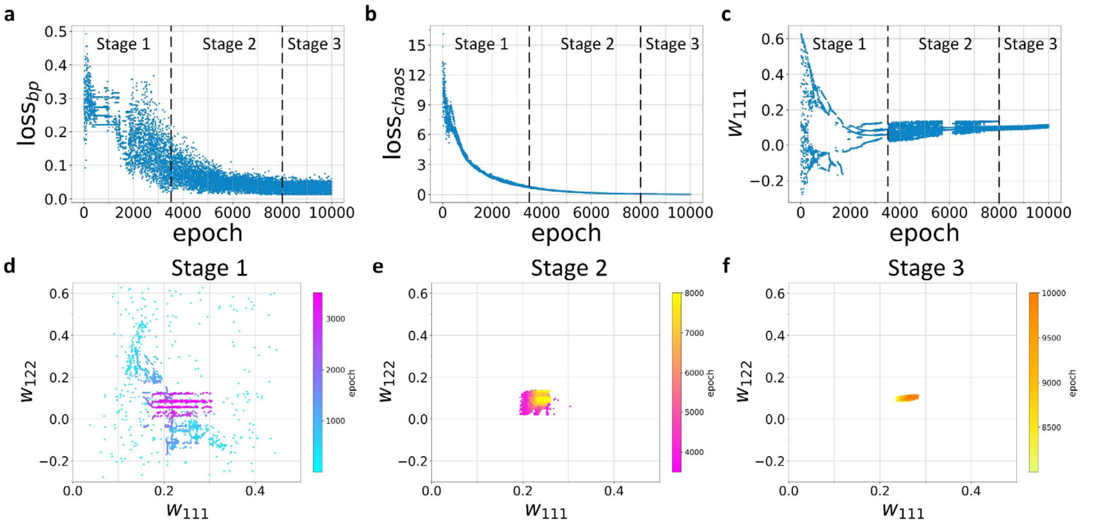

Supplementary Fig. 11. Analysis of the respective roles of loss_{chaos} and loss_{bp} in training a small SNN. **(a-c)** The learning dynamics of loss_{bp}, loss_{chaos} and w_{111}. (**d-f**) When loss_{chaos} is relatively large and dominant (stage 1), loss_{bp} searches a wide weight space; When loss_{chaos} is relatively small after chaotic annealing (stage 2), the search range narrows; As loss_{chaos} approaches 0 (stage 3), loss_{bp} dominates and the dynamics gradually converges. The learning process was conducted on a one-layer SNN with dimensions [10, 3]. Input data and target data were randomly generated from the range [-1, 1]. The simulation time T was set to 10.

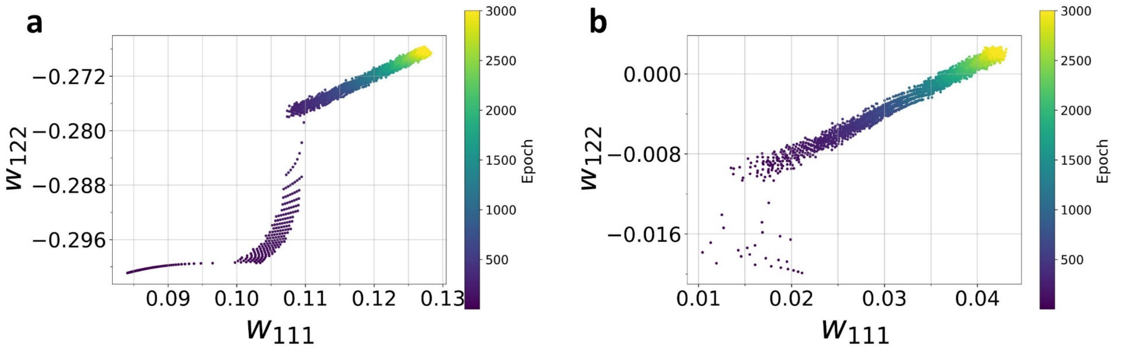

Supplementary Fig. 12. The dynamics of network weight trained by chaotic optimizers with a logistic map under different hyper-parameters **(a)** $r = 3.82$ and **(b)** $r = 3.92$, respectively. The learning process was conducted on a one-layer SNN with dimensions [10, 3]. Input data and target data were randomly generated from the range [-1, 1]. The simulation time T was set to 8.

Supplementary References

[1] L. Chen and K. Aihara, "Global Searching Ability of Chaotic Neural Networks," IEEE Transactions on Circuits and Systems-I, vol. 46, 974-993, 1999.

[2] L. Chen and K. Aihara, "Strange Attractors in Chaotic Neural Networks", IEEE Transactions on Circuits and Systems-I, vol. 47, 1455-1468, 2000.

[3] L. Chen and K.Aihara, "Chaos and asymptotical stability in discrete-time neural networks," Physica D, 104:286-325, 1997.

[4] M. Urabe, "Galerkin's procedure for nonlinear periodic systems," ARCH Rat. Mech. Anal., vol.72, 121-152, 1965.

[5] F.R.Marotto, Snap-back repellers imply chaos in Rn, J. Math. Anal. Appl. 63: 199-223, 1978.

[6] T.Y.Li and J.A.York, Period three implies chaos, Amer. Math. Monthly, 82: 985-992, 1975.

[7] S.-Q. Zhang, Z.-Y. Zhang, Z.-H. Zhou, Bifurcation spiking neural network, the Journal of machine Learning research, 22: 11459-11479, 2021.

[8] Hodgkin AL, Huxley AF. A quantitative description of membrane current and its application to conduction and excitation in nerve. The Journal of physiology. 1952; 117(4): 500.

[9] FitzHugh R. Impulses and physiological states in theoretical models of nerve membrane. Biophysical journal. 1961; 1(6): 445-466.

[10] Nagumo J, Arimoto S, Yoshizawa S. An active pulse transmission line simulating nerve axon. Proceedings of the IRE. 1962; 50(10): 2061-2070.

[11] Nagumo J, Sato S. On a response characteristic of a mathematical neuron model. Kybernetik. 1972; 10(3): 155-164.

[12] Skarda CA, Freeman WJ. How brains make chaos in order to make sense of the world. Behavioral and brain sciences. 1987; 10(2): 161-173.

[13] Matsumoto G, Aihara K, Hanyu Y et al. Chaos and phase locking in normal squid axons. Phys Lett A. 1987; 123(4): 162-166.

[14] Aihara K, Takabe T, Toyoda M. Chaotic neural networks. Physics letters A. 1990; 144(6-7): 333-340.

[15] Korn, H.; Faure, P. Is there chaos in the brain? II. Experimental evidence and related models. C. R. Biol. 2003, 326 (9), 787-840.

[16] Fosque, L. J.; Williams-García, R. V.; Beggs, J. M.; Ortiz, G. Evidence for quasicritical brain

dynamics. Phys. Rev. Lett. 2021, 126 (9), 098101.
